# Indicative bacterial communities and taxa of disease-suppressing and growth-promoting composts and their associations to the rhizoplane

**DOI:** 10.1093/femsec/fiab134

**Published:** 2021-09-21

**Authors:** Johanna Mayerhofer, Barbara Thuerig, Thomas Oberhaensli, Eileen Enderle, Stefanie Lutz, Christian H Ahrens, Jacques G Fuchs, Franco Widmer

**Affiliations:** Molecular Ecology, Agroscope, 8046, Zurich, Switzerland; Crop Protection and Phytopathology, FiBL Research Institute of Organic Agriculture, 5070, Frick, Switzerland; Crop Protection and Phytopathology, FiBL Research Institute of Organic Agriculture, 5070, Frick, Switzerland; Crop Protection and Phytopathology, FiBL Research Institute of Organic Agriculture, 5070, Frick, Switzerland; Molecular Diagnostics, Genomics and Bioinformatics, Agroscope, 8820, Wädenswil, Switzerland; Molecular Diagnostics, Genomics and Bioinformatics, Agroscope, 8820, Wädenswil, Switzerland; Bioinformatics and Proteogenomics, SIB Swiss Institute of Bioinformatics, 8820, Wädenswil, Switzerland; Crop Protection and Phytopathology, FiBL Research Institute of Organic Agriculture, 5070, Frick, Switzerland; Molecular Ecology, Agroscope, 8046, Zurich, Switzerland

**Keywords:** compost microbiome, amplicon sequencing, sequence variant, soil-borne pathogen, targeted strain isolation, diagnostics

## Abstract

Compost applications vary in their plant growth promotion and plant disease suppression, likely due to differences in physico-chemical and biological parameters. Our hypothesis was that bacteria are important for plant growth promotion and disease suppression of composts and, therefore, composts having these traits would contain similar sets of indicative bacterial taxa. Seventeen composts prepared from five different commercial providers and different starting materials were classified accordingly with bioassays using cress plants and the pathogen *Pythium ultimum*. Using a metabarcoding approach, bacterial communities were assessed in bulk composts and cress rhizoplanes. Six and nine composts showed significant disease suppression or growth promotion, respectively, but these traits did not correlate. Growth promotion correlated positively with nitrate content of composts, whereas disease suppression correlated negatively with factors representing compost age. Growth promotion and disease suppression explained significant portions of variation in bacterial community structures, i.e. 11.5% and 14.7%, respectively. Among the sequence variants (SVs) associated with growth promotion, *Microvirga*, *Acinetobacter*, *Streptomyces*, *Bradyrhizobium* and *Bacillus* were highly promising, while in suppressive composts, *Ureibacillus*,*Thermogutta* and *Sphingopyxis* were most promising. Associated SVs represent the basis for developing prediction tools for growth promotion and disease suppression, a highly desired goal for targeted compost production and application.

## Teaser

Bacterial community structures and indicator taxa of composts and rhizoplanes, which correlate with growth promotion or *Pythium ultimum* disease suppression in cress plants.

## INTRODUCTION

In agricultural systems, composts are applied to promote plant growth, suppress soil-borne diseases and improve soil properties like water holding capacity and long-term nutrient content (Agegnehu, Nelson and Bird 2016; De Corato 2020; White *et al*. 2020). Composts are aerobically fermented recycling products of mostly organic raw materials such as plant and food residues and manure, which undergo a thermal and a curing phase leading to stabilization of the organic material and establishment of a characteristic microbiota (Melis and Castaldi 2004; Neher *et al*. 2013). It has been suggested that the compost microbiota plays an important role in plant growth promotion and disease suppression (Antoniou *et al*. 2017; Oberhaensli *et al*. 2017).

Plant growth promotion represents the increase in plant yield resulting, for instance, from compost applications. Such increased yields have been shown in several field trials (Mkhabela and Warman 2005; Pane *et al*. 2015). Growth promotion caused by compost application has been attributed to an increase in available nutrients, e.g. nitrogen and phosphorus, and to an improved water holding capacity (Bonanomi *et al*. 2014; Agegnehu, Nelson and Bird 2016; Redel *et al*. 2021). Besides increased plant biomass due to improved physical and chemical soil properties, compost applications have entailed the introduction of plant growth-promoting bacteria (Carvalhais *et al*. 2013; Antoniou *et al*. 2017). Mechanisms of plant growth promotion by rhizobacteria have been related to an increase of nutrient availability, e.g. due to solubilization of phosphorus or nitrogen fixation, direct stimulation of plant growth due to the production of plant hormones and suppression of diseases (e.g. reviewed in Martínez-Viveros *et al*. 2010). The concept of growth promotion therefore includes disease suppression; however, so far, only few studies have assessed suppression and growth promotion of composts in the same experiment (Antoniou *et al*. 2017; Scotti *et al*. 2020). It is thus not clear whether the same biological, chemical and/or physical compost characteristics are involved in suppression and growth promotion of composts.

Even though the disease suppressive properties of composts have long been recognized and composts have routinely been applied in practice, the unpredictability of suppression of a particular compost remains a major challenge (Noble and Coventry 2005; Bonanomi *et al*. 2007). For instance, 18 tested composts exhibited widely differing disease suppression (between 17% and 94%) in bioassays studying effects of the different plant pathogens *Rhizoctonia solani*, *Phytophthora cinnamomi*, *Phytophthora nicotianae*, *Fusarium oxysporum*, *Verticillium dahliae* and *Cylindrocladium spathiphyllum* (Termorshuizen *et al*. 2006). Therefore, it is likely that microbiological, enzymatic or chemical compost characteristics associated with suppression differ among pathogens, which has also been supported by a metastudy, in which no general predictors of disease suppression were found (Bonanomi *et al*. 2010). Different mechanisms for the role of microorganisms in disease suppression have been proposed (Noble and Coventry 2005; Lutz *et al*. 2020). They include direct effects such as antibiosis, i.e. direct attack of the pathogen and production of antibiotic substances acting against the pathogen as well as successful competition for nutrients. Indirect mechanisms include induction of resistance in the plant and disease resistance by providing nutrients to the plant.

One of the most challenging and devastating soil-borne pathogens is the oomycete *Pythium ultimum*, which causes seedling damping-off, root rots and wilts (Agrios 2005; Raaijmakers *et al*. 2009) and which is characterized by a large host range, including not only cucumber, spinach, peas, soybeans, cotton and maize but also trees and ornamentals. Disease suppression of composts against *Pythium* spp. has previously been related to chemical, physical and, most frequently, biological compost characteristics (Chen, Hoitink and Madden 1988; Craft and Nelson 1996; Scheuerell, Sullivan and Mahaffee 2005; Pane *et al*. 2011). For example, sterilization of composts has been demonstrated to cause loss of disease suppression (e.g. Chen, Hoitink and Madden 1988), and disease suppression against *P. ultimum* and other *Pythium* species has been related to a higher respiration potential of soil to which composts had been applied (Scheuerell, Sullivan and Mahaffee 2005) and to increased microbial activity (Chen, Hoitink and Madden 1988; Craft and Nelson 1996). Using isolation techniques and counts of colony forming units on selective growth media, counts of heterotrophic bacteria and actinomycetes have correlated negatively with disease severity of *P. myriotylum* (Djeugap, Azia and Fontem 2014). In several studies, suppressive activities of bacterial strains isolated from different suppressive environments, such as composts, suppressive soils or rhizospheres of protected plants, have been evaluated *in vitro* for growth inhibition of *Pythium* or in plant–pathogen bioassays for disease suppression (Lutz *et al*. 2020). For example, *Aeromonas media* has been identified as an important component of a compost suppressive against *P. ultimum* (Oberhaensli *et al*. 2017), and *Bacillus* spp., *Pseudomonas* spp. and *Achromobacter xylosoxidans* isolated from compost extracts have been suppressive against *P. aphanidermatum* (Ben Jenana *et al*. 2009). Further, microbial consortia derived from cotton seed surfaces during germination in suppressive composts have been suppressive to *P. ultimum* and their suppressive activities have been stronger than those from individual bacterial isolates (McKellar and Nelson 2003).

For a better understanding of the role of compost microbiota in growth promotion and disease suppression, studies based on highly resolving DNA-based metabarcoding methods are necessary. Such high-throughput sequencing methods have been applied to study microbial compost communities potentially suppressive against *P. ultimum* (Yu *et al*. 2015; De Corato *et al*. 2019). Their application has revealed potentially suppressive taxa at the phylum level (Yu *et al*. 2015) and has been used to search for species known for biocontrol activities, i.e. *Bacillus* spp., *Pseudomonas* spp., *Flavobacterium* spp. and *Trichoderma* spp., among their most abundant taxa (De Corato *et al*. 2019). So far, few such detailed studies on the suppression of composts against *Pythium* spp. have been performed and they lacked a detailed search for taxa at a low taxonomic rank, such as operational taxonomic units (OTUs) or sequence variants (SVs), that are associated with suppression. The importance of considering microbial communities not only in the plant growth medium, e.g. composts or suppressive soils, but also in plant-associated habitats in the context of growth promotion and disease suppression has previously been shown (McKellar and Nelson 2003). Microorganisms, including those involved in disease suppression or growth promotion, as well as pathogens, may be attracted to the spermo- and rhizosphere by seed and root exudates, respectively (reviewed in Mendes, Garbeva and Raaijmakers 2013). Therefore, the area of the root surface, i.e. the rhizoplane, may be the habitat in which microorganism-induced disease suppression or plant growth promotion most likely takes place. Indeed, bacteria from rhizoplanes or rhizospheres have been isolated and successfully tested for disease suppression and/or plant growth promotion (Antoniou *et al*. 2017; Oberhaensli *et al*. 2017).

The aim of the present study was (i) to assess growth promotion and disease suppression of composts using the plant–pathogen system cress and *P. ultimum*, (ii) to analyse the diversity of bacterial communities among different composts and in the rhizoplane of cress plants grown therein, (iii) to assess whether bacterial communities in composts and rhizoplanes correlate with compost characteristics and growth promotion as well as disease suppression against *P. ultimum* and (iv) to determine bacterial taxa, which are more abundant in growth-promoting or suppressive composts and which may potentially be involved in these activities. Therefore, 17 composts from five different professional composting facilities using different starting materials were tested for their ability to promote growth of cress plants and suppress disease symptoms caused by the oomycete *P. ultimum*. Bacterial communities in composts, rhizoplanes and growth matrices were analysed using a metabarcoding approach with respect to their contribution to growth promotion and disease suppression.

## MATERIALS AND METHODS

### Compost characteristics

Seventeen composts were obtained from five different commercial composting facilities. Composts differed in their starting materials, but all contained predominantly plant residues and optionally kitchen waste, manure, humus, mature compost, fibre chalk as well as additives such as biochar or diatomaceous earth (Table S1, Supporting Information). Several physico-chemical parameters of the composts were assessed, such as dry matter, pH, salinity, as well as content of soluble humic substances, ammonia, nitrate and total inorganic nitrogen (Table S2, Supporting Information). Composts were sieved with a mesh size of 10 mm and their dry matter content was determined after heating for 16 h at 105°C. For the assessment of physico-chemical factors, H_2_O and CaCl_2_ extracts were prepared by adding 50 g of compost to either 500 mL of deionized H_2_O or to 500 mL of 0.01 M CaCl_2_ and by agitating on a horizontal shaker at 250 rpm for 1h. The pH of the H_2_O-extracts was determined using a pH meter SevenEasy (Mettler-Toledo, Columbus, OH, USA). The extracts were filtered through cellulose filter paper with a pore size of 2–4 µm (Macherey-Nagel, Düren, Germany). Electrical conductivity of the filtered H_2_O-extract was measured using a FiveGo Conductivity meter (Mettler-Toledo, Columbus, OH, USA). Electrical conductivity was used as proxy for compost salinity (Salinity [KCl_eq_ (kg dry weight)^–1^] = electrical conductivity [mS cm^–1^] × 583.4/dry weight [%]). Content of soluble humic substances of the filtered CaCl_2_ extracts was determined with absorbance at 550 nm (Abächerli *et al*. 2010) (Genesys 150 spectrophotometer, ThermoFisher Scientific, Waltham, MA, USA). Content of ammonia and nitrate of the filtered CaCl_2_ extracts were determined with the Berthelot's reagent for the former and the cadmium-reduction method for the latter (Smartchem 450 Discrete Analyser, AMS Alliance, Guidonia, Italy).

### Bioassay for disease suppression and growth promotion

For the bioassay, the plant-pathogen system cress−*P. ultimum* was chosen for several reasons in addition to *P. ultimum* causing an important economic disease. These included the possibility to assess a large number of composts due to the fast growth of the cress plants, the consistent results for disease suppression and its wide use (Maurhofer *et al*. 1992; Erhart *et al*. 1999; Ishimoto, Fukushi and Tahara 2004; Hunter *et al*. 2006; Thuerig *et al*. 2009; Tamm *et al*. 2010; Pane *et al*. 2011; Bongiorno *et al*. 2019).

Experimental design of the bioassays is displayed in Fig. S1 (Supporting Information). Compost samples, stored at 4°C,  were moistened with water and incubated at 20°C for one week prior to the start of the disease suppression bioassays to allow for reactivation of microorganisms. Three 0.5 mL samples of each compost were taken after the incubation and before the start of the tests and stored at –20°C for subsequent DNA extraction. A 1:1 (v/v) mixture of a unfertilized standard peat substrate (Einheitserde Typ 0, Einheitserdewerke Werkverband e.V., Sinntal-Altengronau, Germany) and vermiculite (0.7–2.0 mm, ISOLA Vermiculite AG, Bözen, Switzerland) was fertilized with 2.3 g L^–1^ horn meal (Biorga Hornmehl, Hauert, Grossaffoltern, Switzerland), moistened with water and amended with 20% (v/v) compost. A peat-vermiculite mix fertilized with 2.3 g L^–1^ horn meal, 0.56 g L^–1^ Thomas phosphate and 1.33 g L^–1^ Kalimagnesium (Biorganic Kali-Magnesia, Hauert, Grossaffoltern, Switzerland) to compensate for input of nutrients by compost application served as a control and was termed ‘control matrix’ in the text.

*Pythium ultimum* was grown on Potato Dextrose Agar (PDA; X931.2, Carl Roth, Karlsruhe, Germany) at 18°C for 6 days. Six agar plugs with a diameter of 5 mm were cut from these agar plates and then added to cooked, sterilized organic millet seeds in sealed cultivation bags equipped with two aeration filter strips (PP75/SEU2/V18.7–32, SacO2, Nevele, Belgium). After one week, *P. ultimum–*millet mix was added to water [20% (w/v)] and homogenized with a disperser (Ultra Turrax T25, IKA^®^-Werke GmbH & CO. KG, Staufen, Germany) at 8000 rpm for 30 s. Serial dilutions of the *P. ultimum–*millet suspension were prepared with water and mixed with sand to allow for a homogeneous distribution in the substrate. Twenty g L^–1^ of *P. ultimum–*millet–sand mixtures containing different quantities of *P. ultimum* were added to substrates to obtain final concentrations of 0.25, 0.5 and 1 g L^–1^ of the *P. ultimum–*millet mix in the growth substrates, i.e. compost substrate and control matrix.

Compost screening bioassays were performed in 300-cell seedling trays with a cell volume of 25 mL. Six adjacent cells were used for each of the three replicates per treatment. Replicates were positioned randomly in a chequered pattern with empty wells in between to avoid physical contact among treatments. On average, 190 organic cress seeds (*Lepidium sativum*, Bigler Samen AG, Steffisburg, Switzerland) were sown per replicate with a sowing spoon and moistened with a hand sprayer. Trays were covered with plastic foil and plants were cultivated in growth chambers with a 16/8 h day–night cycle at a light intensity of 130 µmol m^–2^ s^–1^. After a germination period of 2 d at 100% relative humidity, the plastic foil was removed and the cress was grown at 66% relative humidity. Plants were watered by flooding and draining every two to three days. At the end of the bioassay, i.e. after six to seven days, plants were cut above the soil surface and shoot fresh weight was measured for each replicate pot. Cress shoot weight was used to calculate % growth promotion and disease suppression. Using the relative data instead of cress shoot weight, enabled the separation of the two traits as follows. Growth promotion (in %) was calculated by dividing the biomass of cress grown in a compost substrate (m_C_) by the mean cress biomass grown in the control matrix (m_M_), both without inoculation with the pathogen. Values were adjusted by subtracting 100, to obtain 0% growth promotion for the control matrix, i.e. m_C_ equals m_M_, [(m_C_/m_M_ × 100) – 100]. Values may result in a minimum of –100% and the maximum was unrestricted. As a measure for disease suppression, the shoot weight of each *P. ultimum*-inoculated sample (m_PU_) was divided by the mean of the three corresponding uninoculated samples (m_UI_) for each compost and the control matrix [m_PU_/m_UI_ × 100] yielding 100% disease suppression if m_PU_ equals m_UI._ The minimum possible value of % disease suppression was 0% and the maximum was unrestricted. In a control assessment disease symptoms of damping-off caused by *P. ultimum* were classified in five categories with increasing severity of disease symptoms, i.e. yellowing of leaves and reduction of growth. The scores of disease symptom classes were: ‘0’ no symptoms, ‘1’ few symptoms, ‘2’ medium symptoms, ‘3’ strong symptoms and ‘4’ no germination at all. Data on cress shoot weight and disease symptoms is provided in Table S3 (Supporting Information). Medians of disease symptom scores were strongly negatively correlated with mean values of mean disease suppression including all levels of inoculation with *P. ultimum* (Spearman rho = –0.88, *P* < 2.2 × 10^–16^). Therefore, the shoot weight reflects very well the disease symptoms caused by *P. ultimum*. Furthermore, cress shoot fresh weight and relative data have been used as a measure to describe disease severity in several studies (Maurhofer *et al*. 1992; Erhart *et al*. 1999; Thuerig *et al*. 2009; Tamm *et al*. 2010; Bongiorno *et al*. 2019). This is possible since in cress, *P. ultimum* mainly causes a pre-emerging damping-off and therefore reduces the number of seedlings. Furthermore, the remaining seedlings are often stunted and might die after a prolonged period. Therefore, we used the shoot weight-based values to describe disease suppression throughout this study.

### Harvest of the rhizoplane

Rhizoplanes were harvested at the end of the bioassay. Soil particles were removed from the roots by shaking and rinsing with water. After weighing, cleaned fresh roots were placed in 50 mL Falcon tubes containing 20 mL 0.8% NaCl solution, and stored on ice until further processing. Roots in the solution were transferred to 100 mL Erlenmeyer flasks. Eight grams of glass beads with a diameter of 3 mm (Huber Lab, Aesch, Switzerland) were added to each flask, which were then agitated at 300 rpm for 15 min on a horizontal shaker. The solutions were filtered into 50 mL Eppendorf tubes using a fleece filter with a pore size of ∼100 µm (Type FT25, Sana, Switzerland) in order to remove root particles and beads. The filtered solutions were centrifuged using a swing-out rotor (2K-15 centrifuge, Sigma, Osterode am Harz, Germany) at 2500 g and 4°C for 20 min. Subsequently, the pellet was resuspended in 1 mL 0.8% NaCl solution and stored at –20°C until DNA extraction.

### DNA extraction of composts and rhizoplanes

Total DNA was extracted from three replicates of each compost and rhizoplane (Fig. S1, Supporting Information). For each compost sample, ∼0.5 mL of compost were measured into a 2 mL Eppendorf tube and DNA was extracted following the protocol of the NucleoSpin 96 soil extraction kit (Macherey-Nagel, Düren, Germany). For lysing microbial cells in the compost, the SL1 buffer and the SX enhancer solution of the kit were used and the lysis was performed using a TissueLyser (Qiagen, Hilden, Germany) at 30 s^–1^ for 4 min. Extraction of DNA from the rhizoplane was performed using the Quick-DNA Fungal/Bacterial Miniprep kit (Zymo Research, Irvine, CA, USA). DNA content of all extracts was determined using the Quant-iT PicoGreen dsDNA Assay kit (Invitrogen, Waltham, MA, USA) using a Cary Eclipse fluorescence spectrophotometer (Varian, Palo Alto, CA, USA). DNA extracts were diluted to 5 ng μL^–1^ using deionized and autoclaved H_2_O.

### 16S rRNA marker gene analysis

The variable region V3-V4 of the 16S rRNA gene marker was amplified with the primer pair 341F/806R, which was modified by Frey *et al*. (2016). Briefly, each polymerase chain reaction (PCR) included 20 ng DNA, 0.2 µM of each primer, 0.2 mM dNTPs (Promega, Madison, WI, USA), 2.5 mM MgCl_2_, 0.6 mg mL^–1^ bovine serum albumin, GoTaq Flexi buffer (Promega, Madison, WI, USA) and 1.25 units of the GoTaq Hotstart G2 polymerase. Deionized and autoclaved H_2_O was added to a final volume of 25 µL. PCR was performed in 96-well plates and in each plate one negative control was included, which contained all reagents except replacing DNA with water. PCR cycling conditions consisted of an initial denaturation step for 2 min at 95°C,  followed by 35 cycles of denaturation for 40 s at 94°C,  annealing for 40 s at 58°C and elongation for 1 min at 72°C. The PCR was finalized by elongation at 72°C for 10 min. PCR of each sample was independently performed four times and subsequently pooled. Quality of the PCR products was checked using Agarose gel electrophoresis. Multiplexing of the PCR products was enabled by the primer adapters CS1 and CS2 of the Fluidigm Access Array System (Fluidigm, San Francisco, CA, USA). Paired-end sequencing was performed on the Illumina MiSeq v3 platform (Illumina, San Diego, CA, USA) at the Genome Quebec Innovation Centre (McGill University, Québec, Canada).

### Quality control and bioinformatic analyses

Quality control of the raw sequences was performed using a bioinformatic pipeline based on UPARSE (Edgar 2013) and MOTHUR v1.39.5 (Schloss *et al*. 2009) as described by Mayerhofer *et al*. (2021). Except, sequences were de-noised to obtain SVs using UNOISE v2 (Edgar 2016) instead of clustering them into OTUs with a 97% identity threshold. The aim of obtaining SVs is to increase the resolution of amplicon sequence analysis. Taxonomic classification of SVs was performed using the SILVA database release 132 (Quast *et al*. 2013) with the command ‘classify.seqs’ in MOTHUR. SVs that were classified as Archaea or organelles, i.e. chloroplasts or mitochondria, were removed from the dataset. Raw sequence data are available at the SRA database (PRJNA725376).

### Statistical analyses

Statistical analyses were performed in Rstudio version 1.3.1093-1 and R version 4.0.2 (R-Core-Team 2020) unless otherwise specified. Differences of univariate data among groups were obtained using one-way analysis of variance (ANOVA) followed by Tukey's honestly significant difference (Tukey HSD) post-hoc tests with the R-function ‘emmeans’ (Lenth 2020), and Pearson and Spearman correlations between univariate variables were performed using R core. SV richness and coverage of bacterial communities were determined using ‘summary.single’ in MOTHUR (Schloss *et al*. 2009), which includes a subsampling procedure to the lowest number of sequences of a sample (i.e. 13 173 sequences) with 10 000 iterations. Bacterial community structures were calculated based on Bray–Curtis dissimilarities either based on relative SV abundances using ‘vegdist’ in the R-package ‘vegan’ (Oksanen *et al*. 2019) or by subsampling to the lowest number of sequences with 10 000 iterations using the function ‘dist.shared’ in MOTHUR. Dissimilarity matrices of the two methods were highly correlated (Mantel test in ‘vegan’; Pearson *r* = 1.00, *P* = 0.0001), enabling the use of Bray–Curtis dissimilarities based on relative abundance for the following analyses. Permutational multivariate analyis of variance (PERMANOVA) was used to test for differences of bacterial community structures among substrates or among rhizoplanes grown in the same substrate using the function ‘adonis’ in the package vegan. The same function was used to assess correlations of mean growth promotion as well as disease suppression with mean bacterial community structures, for which Bray–Curtis dissimilarities were calculated based on mean relative SV abundances including only robustly detectable SVs, i.e. which occurred in at least two replicates of a substrate or of rhizoplanes grown in the same substrate. Robustly detected SVs were also used for partitioning SVs among compost substrates, the control matrix and rhizoplanes and for assessing associations of SVs to growth promotion and disease suppression. Partitioning of SVs to the different compartments, i.e. substrates and rhizoplanes treated with substrates with or without inoculation with the pathogen, was performed with the function ‘draw.quadruple.venn’ in the package ‘VennDiagram’ (Chen 2018). This was performed for each compost and included a subsampling procedure to the lowest number of sequences of a sample with 100 iterations. Partitioning was also performed for the five most and least growth-promoting as well as disease-suppressing composts and the Student *t*-test was used to identify areas of the Venn diagrams with significantly different percentages of SVs. Associations of robustly detected SVs to composts with strong plant growth promotion and disease suppression were calculated with an indicator species analysis using the function ‘multipatt’ with the R-package ‘indicspecies’ (De Cáceres and Legendre 2009). For that, the association function ‘r.g’, which relies on the point biserial correlation coefficient, was used.

## RESULTS

### Plant growth promotion of composts

**Figure 1.**
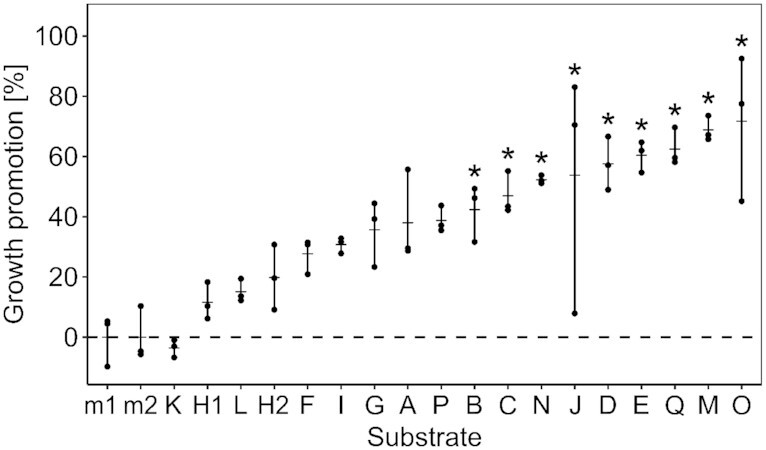
Growth promotion of cress plants due to compost applications. Percent growth promotion was measured as % increase in cress shoot weight compared with the matrix within each batch. Control (m1 and m2) and compost treatments (A–Q) were sorted in ascending order by their mean % growth promotion, and compost H was tested in both batches (H1 and H2) to assess the interbatch variability. Dashes and dots represent means and values of the replicates of each substrate, respectively. Asterisks indicate significant differences of compost treatments compared with both control matrices (Tukey HSD tests, *P* < 0.05). The dashed line shows 0% growth promotion, which corresponds to the mean cress biomass of the matrix (m1 and m2).

Plant growth promotion was recorded as the % difference in shoot weight of plants that were grown in compost substrates compared with the fertilized control matrix, both without inoculation with *P. ultimum*. The experiment contained two consecutive batches of nine composts, which both included compost H to estimate the interbatch variability (composts A to I and J to Q plus H). The growth-promoting effect of compost H, which was tested in both batches, did not differ significantly among the batches (*P* = 1.000). Therefore, the batches were analysed together. There was a significant difference of growth promotion among growth substrates (ANOVA F = 10.1, *P* = 6.8 × 10^–10^; Fig. 1). Nine of the 17 composts showed a significantly higher mean shoot weight than the control and their mean increase ranged from 42 to 72%. However, composts J and O showed a large variance of growth promotion; therefore, the data were less reliable. Correlations of growth promotion with the compost characteristics age, dry matter, pH, salinity, content of soluble humic substances, nitrate, ammonia and total inorganic nitrogen, excluding the control matrix from the calculations, showed that mean growth promotion was only significantly correlated with nitrate (rho = 0.52, *P* = 0.034, *n* = 17; Fig. S2 and Table S4, Supporting Information).

### Compost treatment-associated suppression of *P. ultimum* caused plant disease

**Figure 2.**
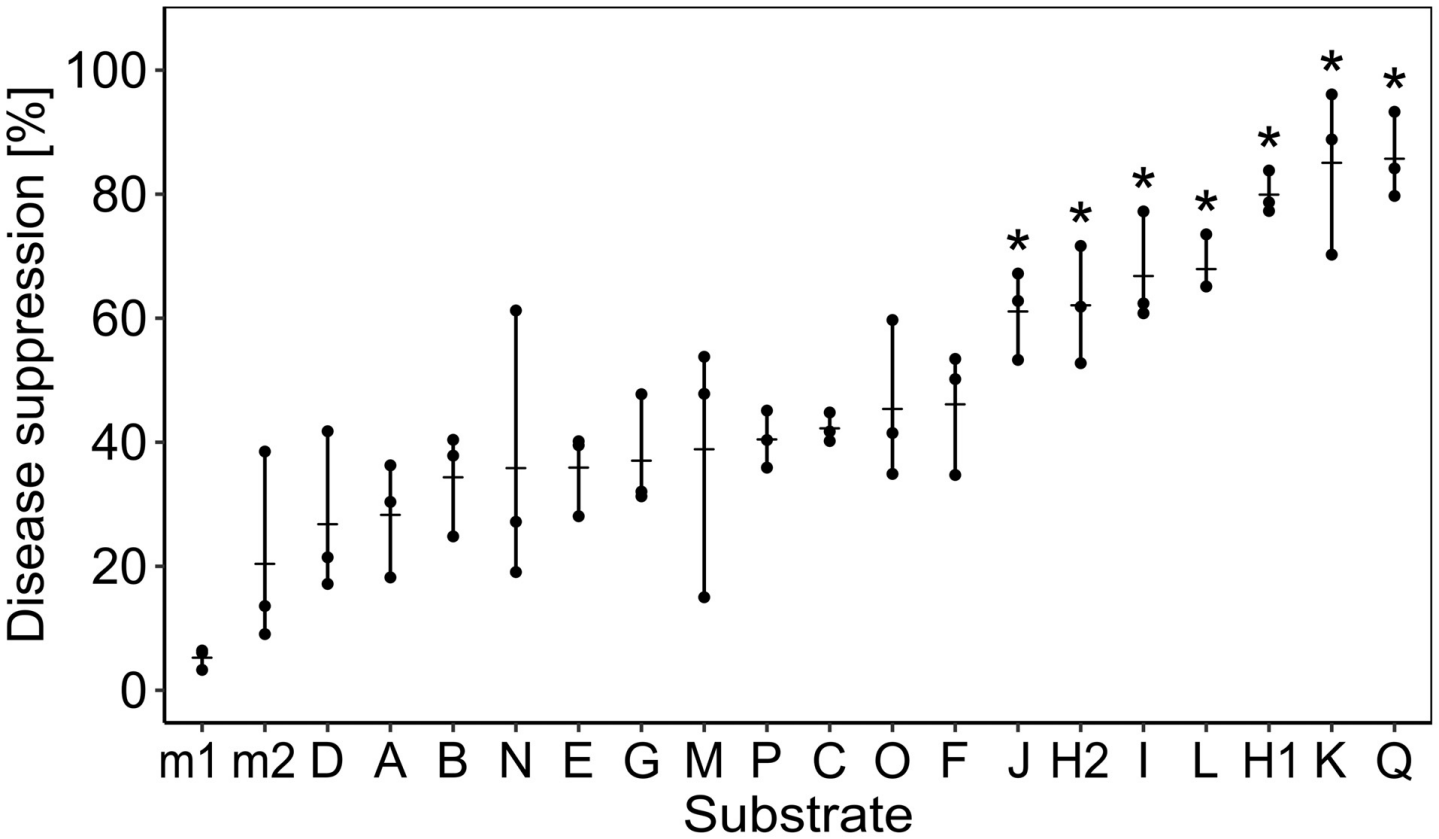
Disease suppression against *P. ultimum* of compost applications. Disease suppression was defined as the relative shoot weight of *Pythium*-inoculated compared with -uninoculated plants and calculated for each of the compost substrates and the control matrix in two batches. Control (m1 and m2) and compost treatments (A–Q) were ordered by their mean % disease suppression, and compost H was tested in both batches (H1 and H2) to assess the interbatch variability. Dashes and dots represent means and values of the replicates of each treatment. Asterisks indicate significant differences of compost treatments compared with both control matrices (Tukey HSD tests, *P* < 0.05).

Disease suppression, i.e. the relative shoot weight of *P. ultimum*-inoculated compared with -uninoculated cress plants, was determined for the 17 composts and the control matrix in the same two consecutive batches as described above. In each batch, the composts were tested with different inoculation quantities of the pathogen, i.e. 0.25, 0.5 and 1 g of *P. ultimum*–millet mix L^–1^ substrate (Fig. S3, Supporting Information). At 1 g L^–1^, the inoculum lead to a pronounced decrease of plant growth in the untreated control matrix, and therefore this concentration was selected for characterizing disease suppression of each compost and further analyses. Compost H, which was used in both batches, showed a mean disease suppression of 80% and 62% in the two batches, with no significant difference (*P* = 0.890). Similarly, mean disease suppression of the control matrix was 5% and 20% for the two batches, again with no significant difference between them (*P* = 0.972). Therefore, we combined the two batches. Disease suppression differed significantly among growth substrates, i.e. different compost substrates and control matrix including both batches (ANOVA F = 11.9, *P* = 5.3 × 10^–11^; Fig. 2). Six composts differed significantly from the control matrix with values between 61% and 86% disease suppression (Fig. 2).

Mean disease suppression was significantly negatively correlated with nitrate (rho = –0.56, *P* = 0.019, *n* = 17), total inorganic nitrogen (rho = –0.54, *P* = 0.026, *n* = 17) and compost age (rho = –0.75, *P* = 0.005, *n* = 12), whereas dry matter, pH, salinity and content of soluble humic substances did not show a significant correlation (Fig. S4 and Table S4, Supporting Information). Two composts, i.e. composts J and Q, resulted in both significant growth promotion and disease suppression, while 11 composts showed significant activities in only one of the traits, and four composts, i.e. composts A, F, G and P, had no significant activities. For instance, the second most suppressive compost K was the least growth-promoting compost (Figs 1 and 2). Overall, disease suppression and growth promotion were not significantly correlated (rho = −0.34, *P* = 0.178; Table S4, Supporting Information).

### Metabarcoding sequence analysis

In total, the 16S rRNA barcode was PCR amplified from 174 samples (Fig. S1, Supporting Information). These included 51 samples from composts (3 replicates of 17 composts), 3 from the control matrix and 120 from rhizoplanes of plants grown in compost substrates or control matrix with or without inoculation with *P. ultimum* (3 replicates for each treatment; Fig. S1, Supporting Information). Due to the similarity of 16S rRNA sequences of plant organelles (chloroplasts and mitochondria) and bacteria, they were amplified with the primer pair used (Table S5, Supporting Information), but subsequently excluded from the dataset. On average, 0.006% and 0.01% of the sequences in the compost, 0.003% and none of the sequences in the matrix and 3.5% and 1.3% of the sequences in the rhizoplanes of cress plants grown in compost substrates and 7.9% and 2.9% of the sequences in the rhizoplane of plants grown in the untreated control were assigned to chloroplasts and mitochondria, respectively. These low percentages suggest negligible influence of the presence of plant organelles on the coverage of bacteria during PCR and sequencing. After quality control and removal of nonbacterial sequences 5 038 778 bacterial sequences with a mean of 28 958 (SD 4418) per sample were obtained. A minimum of 13 173 and a maximum of 39 817 sequences were obtained per sample. Sequences were grouped into 23 885 SVs. An SV was considered robustly detected if it occurred in at least two replicates within, i.e. a compost, the control matrix or rhizoplanes of plants grown in the same substrate. Filtering yielded 18 868 SVs. The SV coverage for each sample, i.e. Good's coverage, a measure to estimate the sampling success in terms of completeness, ranged from 87.0 to 96.8% with a mean of 92.7% (SD 1.8%), suggesting that the bacterial communities were well reflected in the sequencing dataset.

### Bacterial communities in substrates

**Figure 3.**
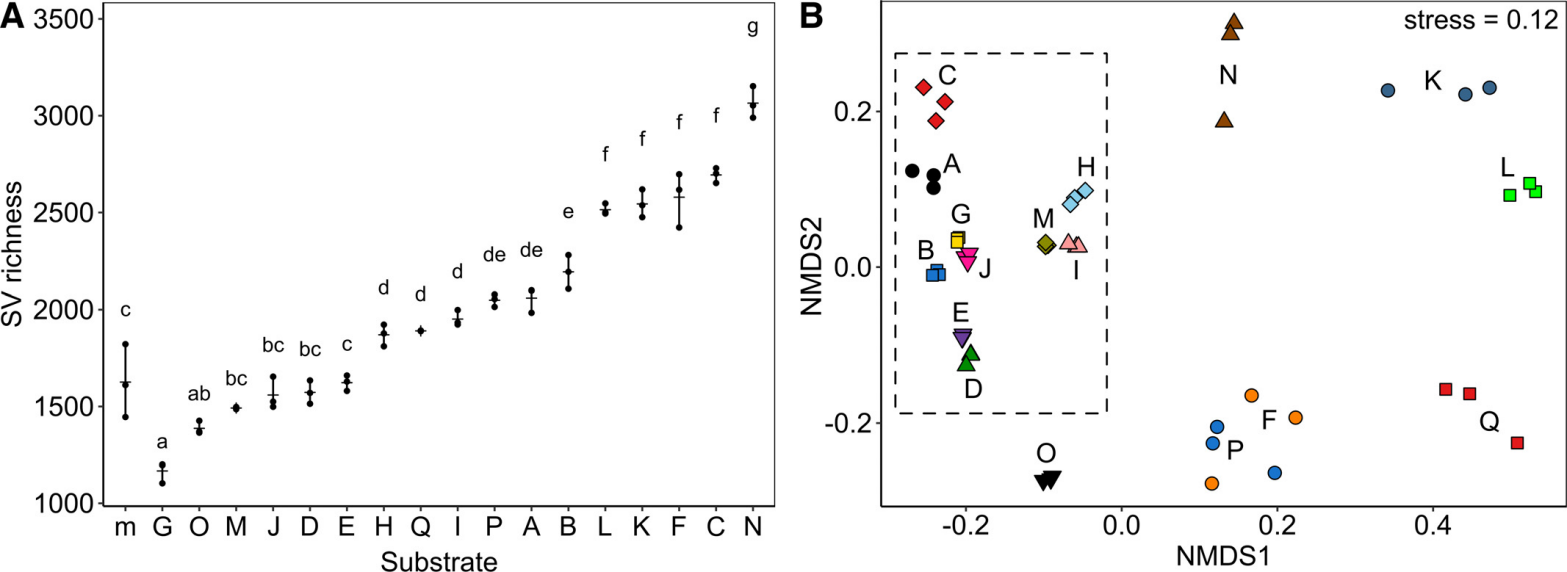
Bacterial SV richness **(A)** of the 17 composts (A–Q) and the matrix (m) and bacterial community structures among composts **(B)**. Compost treatments were ordered by their mean SV richness (A). The dots represent replicates connected by a vertical line and the horizontal dash represents the mean. Lowercase letters indicate significant differences obtained from pairwise Tukey HSD tests with a *P*-value below 0.05. The symbols and colours correspond to composts (A–Q) and the ordination (NMDS) was based on Bray–Curtis dissimilarities (B).

Richness of bacterial SVs differed significantly among composts and the control matrix (ANOVA F-statistic = 139.8, *P* < 2 × 10^–16^) and pairwise comparisons revealed that SV richness was significantly higher in 11 and lower in 2 composts compared with the control matrix. Mean SV richness was 1626 in the control matrix and ranged between 1167 and 3065 in the composts (Fig. 3A). Despite the large differences in SV richness among some composts, there was no significant correlation of mean SV richness neither with mean growth promotion (*r* = –0.44, *P* = 0.076) nor with mean disease suppression (*r* = 0.14, *P* = 0.583). Bacterial community structures differed significantly among composts and the control matrix (PERMANOVA pseudo-F = 36.7, *P* = 0.0001; Table S6, Supporting Information). Differences of bacterial community structures among the 17 composts is displayed on nonmetric multidimensional scaling (NMDS) ordination (Fig. 3B; PERMANOVA pseudo-F = 34.8, *P* = 0.0001). The distinctness of the bacterial communities among the 17 composts was also shown by the small numbers of robustly detected SVs that were shared among different composts, i.e. 16.1% of the SVs occurred only in one compost, 48.3% of the SVs occurred in only up to three composts and only 1.2% occurred in all composts (Table S7, Supporting Information). The ordination revealed a cluster of ten composts, i.e. compost A, B, C, D, E, G, H, I, J and M (Fig. 3B, box with dashed line). For the first eight of them, data on starting materials for the composting process was available and their starting materials included between 5% and 35% manure and between 8% and 10% mature compost (except compost H included only mature compost). In contrast, the remaining compost with no obvious clustering on the ordination contained neither manure nor compost but mostly plant residues among their starting materials (compost F, O, P and Q) or data were not available (compost K, L and N).

Linear models of bacterial community structures with growth promotion and disease suppression revealed that mean growth promotion and mean disease suppression explained significant portions of the variation of bacterial community structures of the 17 composts, i.e. 11.5% and 14.7% respectively (Table S8, Supporting Information). Compost characteristics that were significantly correlated with growth promotion or disease suppression, i.e. nitrate, total inorganic nitrogen and age, were also correlated with bacterial community structures. Nitrate, which was positively correlated with growth promotion and negatively correlated with disease suppression, explained a significant portion of 11.6% of the variation in bacterial community structures, while the correlation with total inorganic nitrogen was only close to significant (*R*^2^ = 10.4, *P* = 0.0698). Age explained 17.6% of the variation in bacterial community structures. However, data on compost age were only available for 12 composts impairing a direct comparison of the effect of compost age with the effects from the other factors for which 17 composts were available. When nitrate was included as a covariate in the model, growth promotion did not add any significantly explained variation. Taking into account nitrate, disease suppression explained an additional 9.5% but the *P*-value was only close to significant (0.0756).

### Partitioning of SV between compost, control matrix and the rhizoplanes

**Figure 4.**
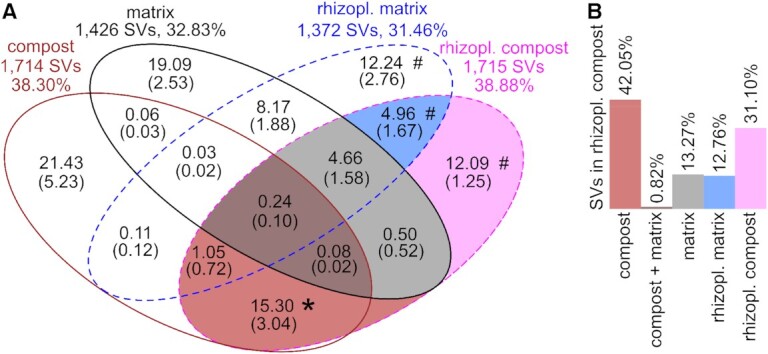
Partitioning of robustly detected SVs among substrates and rhizoplanes. **(A)** Venn diagram displaying the occurrence of SVs in each compartment, i.e. compost (brown line), matrix (black line), rhizoplanes of cress plants grown in compost substrate (pink dashed line) and rhizoplanes of cress plants grown in the matrix (blue dashed line). Rhizoplane data derived from plants not inoculated with *P. ultimum* are presented. A similar visualization for rhizoplane samples of plants inoculated with the pathogen is shown in Fig. S5 (Supporting Information). The numbers outside of the circles of the Venn diagram show the number of SVs and the mean percentage of SVs in each compartment. The numbers in the different areas of the Venn diagram correspond to mean percentages of SVs from the 17 composts, the matrix, the corresponding rhizoplanes and their intersections. Standard deviations are presented in parenthesis. Data were obtained using a subsampling procedure to 13 173 sequences with 100 iterations. The star represents compost-derived rhizoplane bacteria analysed in the 'Bacterial communities in rhizoplanes' section and the hash tag represents SVs in rhizoplanes that were derived neither from the compost nor the matrix. **(B)** Partitioning of SVs in rhizoplanes of plants grown in compost substrates. Bars represent coloured areas in Fig. 4A. Percentages correspond to SVs that were also found in compost, in compost and the matrix, the matrix and in rhizoplanes of plants grown in the control matrix, or that were only detected in rhizoplanes of plants grown in the compost substrates.

Partitioning of bacterial communities among compost, control matrix and rhizoplane of cress plants was assessed for the 18 868 robustly detected SVs (Fig. 4). Partitioning was performed separately for rhizoplanes of pathogen-inoculated (Fig. S5, Supporting Information) and uninoculated plants (Fig. 4), which revealed highly similar results (*r* = 0.98, *P* = 9.1 × 10^–11^). Partitioning was performed for each compost, yielding mean percentages for each area in the Venn diagram and the bar plot, and included a subsampling to accommodate for differences in sequencing depth. On average, 38.30% of the SVs were found in compost (brown line in Fig. 4A) and almost half of them (16.67%) were found in composts and the rhizoplanes of cress grown in compost substrates (intersection of brown and pink line). In comparison, 32.83% of all SVs occurred in the control matrix (black line in Fig. 4A) and only 5.48% were detected in the control matrix as well as in rhizoplanes of plants grown in compost substrates (intersection of black and pink line). There were SVs that were not detected in the substrates (compost and control matrix), i.e. means of 12.24%, 12.09% and 4.96%, and that were found only in the rhizoplanes of the control plants, rhizoplanes grown in compost or in both of them (Fig. 4A; hash tag). These represent SVs that were introduced either with the plant seeds, during handling of the experiments, or grew from taxa that were undetectable in substrates. We focused on SVs of rhizoplanes grown in compost substrates, which were represented by the pink line in Fig. 4A and the bar plot in Fig. 4B. On average 42.05% of the SVs in the rhizoplanes grown in compost substrates were derived from the compost (brown bar) and only 13.27% from the control matrix (grey bar) and 0.82% from both (dark brown bar; Fig. 4B).

Partitioning of SVs was also compared among the five most and least growth-promoting composts (Fig. S6A, Supporting Information) as well as the five most and least disease-suppressing composts (Fig. S6B, Supporting Information). Percentages of numbers of SVs in the areas of the Venn diagrams were not significantly different, except for disease suppression in the area combining control matrix and rhizoplanes of cress plants, i.e. 0.35% and 0.71% in most and least suppressive composts (*P* = 0.038; Fig. S6B and Table S9, Supporting Information). This indicated stable distribution of SVs among the compartments, i.e. compost, matrix and the rhizoplanes of cress plants, regardless of the level of the composts’ activity in growth promotion and disease suppression.

### Bacterial communities in rhizoplanes

The comparison of bacterial communities in composts and in rhizoplanes of cress plants revealed that mean SV richness of the composts correlated significantly with mean SV richness in the rhizoplanes of plants grown in compost substrates in the presence of the pathogen (*r* = 0.68, *P* = 0.002) and in its absence (*r* = 0.87, *P* = 6.7 × 10^–6^). Bacterial community structures of the rhizoplanes differed among the substrates they were grown in, i.e. different composts and the control matrix, independent of inoculation with *P. ultimum* (PERMANOVA, pseudo-F = 6.3, *P* = 0.0001) or not (pseudo-F = 7.3, *P* = 0.0001). Furthermore, the bacterial community structures among the different composts and the control matrix were more distinct than the community structures in the rhizoplanes of plants grown in different growth substrates, which was revealed by a larger pseudo-F value of 36.7 compared with 6.3 and 7.3 in the respective PERMANOVAs (Table S6, Supporting Information). This comparison showed that rhizoplane communities were more similar to each other than compost communities. The rhizoplane communities of plants grown in compost substrates included SVs that originated from composts (Fig. 4A brown area; 4B brown bar), the control matrix (Fig. 4A grey area; 4B grey bar) and rhizoplane-specific SVs (Fig. 4A pink and blue areas, 4B pink and blue bars). To search for compost-derived and potentially growth-promoting and/or disease-suppressive bacteria in the rhizoplanes, we focused on SVs that occurred in the compost and in the rhizoplanes of plants grown in compost substrates, but which were not detected in other samples (Fig. 4A; star). This resulted in a removal of 7509 and 7661 SVs for uninoculated and inoculated communities, respectively. The remaining 11 359 SVs from uninoculated communities and 11 207 SVs from inoculated communities were termed compost-derived rhizoplane SVs. Based on these compost-derived rhizoplane communities, mean growth promotion explained a small portion of the community structures without pathogen inoculation with no statistical support (9.1%, pseudo-F = 1.5, *P* = 0.071; Table S8, Supporting Information). Mean disease suppression explained a small but significant part of 10.8% of the variation of compost-derived rhizoplane community structures in the presence of *P. ultimum* (pseudo-F = 1.8, *P* = 0.021; Table S8, Supporting Information). These effect sizes for growth promotion (9.1%) and disease suppression (10.8%) were slightly smaller than those obtained from analyses including compost community structure (11.5% and 14.7%; see the section 'Bacterial communities in substrates'; Table S8, Supporting Information). As a consequence, compost bacterial communities were screened for SVs that were potentially associated with growth promotion or suppression.

### SVs associated with growth-promoting composts

The 17 composts revealed gradual values for growth promotion and disease suppression without distinct clusters. Therefore, to identify SVs, which are consistently more abundant in composts with high values of these two traits, SVs found in the five composts with the strongest and the weakest plant growth promotion or disease suppression were compared. Indicator species analyses with a point biserial correlation coefficient greater than 0.7 were used as the definition for strongly associated SVs, which are potentially involved in or indicative for growth promotion or disease suppression. To determine SVs indicative for growth promotion (compost J and O were omitted due to their high variability), the most growth-promoting composts D, E, M, N and Q (52–72%) and the least growth-promoting composts F, H, I, K and L (–4 to 31%; Fig. 2) were used. In total, 28 SVs were indicative for growth promotion (Table S10, Supporting Information). Together, the 28 SVs included 0.5% of the relative sequence abundance of all compost samples. Tracking their abundance in the rhizoplanes and the control matrix revealed that all except two SVs were also present in the rhizoplanes grown in growth-promoting compost substrates. In addition, four SVs were also detected in the community of the control matrix and two of them, SV-31 (classified as *Bradyrhizobium* sp.) and SV-13 305 (assigned to the order Rhizobiales), revealed a higher mean abundance in the control matrix than in the growth-promoting composts making their involvement in growth promotion less likely. Nine of the 28 SVs could be taxonomically classified at the genus level comprising *Hyphomicrobium*, *Bradyrhizobium*, *Brevundimonas*, *Streptomyces*, *Microvirga*, *Bacillus*, *Peredibacter*, *Acinetobacter* and *Panacagrimonas*. For the SVs whose taxonomic classification revealed a genus-level identification, a literature search was performed to identify genera, which include species isolated from compost or involved in growth promotion (Table S10, Supporting Information). Seven of them have been previously detected in composts and six of them have been described to play a role in plant growth promotion (for references, see Table S10, Supporting Information). To test the importance of the 28 SVs for growth promotion in all 17 composts (including intermediate growth-promoting composts, i.e. compost A, B, C, G, J, O and P), Spearman correlation of mean growth promotion with each SV was performed for the 10 SVs with an occurrence in 11 to 17 composts. This threshold was set, to ensure the occurrence in more composts than the five least and five most growth-promoting ones. Of the eleven SVs, three revealed significant positive correlations (Table S10, Supporting Information) and these were SV-2097 (belonging to the family Bacillaceae), SV-1147 (classified as *Hyphomicrobium*) and SV-5145 (belonging to the phylum Chloroflexi), corroborating their contribution to growth promotion or indication of growth-promoting composts.

### SVs associated with strongly suppressive composts

To identify SVs potentially involved in disease suppression or revealing indicative properties, bacterial SVs in compost H, I, K, L and Q, which showed the highest disease suppression (67–86%), and compost A, B, D, E and N, which showed the weakest disease suppression (27–36%), were compared. In total, 75 SVs revealed strong associations with strongly suppressive composts (Table S11, Supporting Information). Together, the 75 SVs accounted for a low relative overall sequence abundance of 3%, however, they included a high taxonomic diversity, i.e. at least 24 different genera from 10 phyla (Table S11, Supporting Information). The three most strongly associated SVs were SV-437, SV-48 and SV-1095, which were classified as unknown of the class OLB14 of the phylum Chloroflexi, as unknown of the order Rhizobiales (Proteobacteria) and as unknown of Gammaproteobacteria. Most interestingly, three of the twelve most strongly associated SVs were classified as *Ureibacillus* sp.

Of the associated SVs, 27 were identified at the genus level and they yielded 24 different genera (Table 1). All of these 27 SVs, except for SV-19508 (classified as *Bdellovibrio*), were also detected in the rhizoplanes of cress plants treated with the highly suppressive composts at similar abundances. Furthermore, only SV-64 (classified as *Algoriphagus*) was also detected in the control matrix, however, with 82.7 times lower mean abundance (0.0011%) than in the suppressive composts (0.091%). For the 24 genera, a literature search was performed on occurrence in compost and disease suppression (Table S11, Supporting Information). While 18 genera were reported in studies on compost bacteria, only five genera were mentioned in studies on disease suppression, i.e. *Flavobacterium*, *Pedobacter*, *Rheinheimera*, *Steroidobacter* and *Sphingopyxis* (for references, see Table S11, Supporting Information).

**Table 1.** Relative abundance and taxonomy of 27 SVs, which were associated with strongly suppressive composts, i.e. compost H, I, K, L and Q and which were classified to genus level. Disease suppression was defined as the relative shoot weight of *Pythium*-inoculated compared with -uninoculated plants and calculated for each of the compost substrates and the control matrix. SVs were identified using indicator species analyses based on a point biserial correlation coefficient (*r*_PB_) larger than 0.7. The order of the SVs corresponds to the strength of the associations. Correlations of disease suppression and SVs included all composts, in which the SV was present. References to literature on compost bacteria and disease suppression are included in Table S11 (Supporting Information).

		Cor. incl. all comp.^b^			Mean sequence abundance [%] in				
SV	*r*_PB_^a^	rho	*P*	*n*	Suppr. comp. ^c^	Rhizopl. in suppr. comp.^d^	Matrix	Phylum	Genus
SV-1471	0.89	0.65	0.017	17	6.40E-02	4.20E-02	0	Firmicutes	*Ureibacillus*
SV-2226	0.87	0.64	0.017	11	1.20E-02	1.10E-02	0	Firmicutes	*Ureibacillus*
SV-11381	0.83		NA		5.80E-03	4.20E-03	0	Planctomycetes	*Thermogutta*
SV-458	0.82	0.64	0.017	17	8.60E-02	5.30E-02	0	Firmicutes	*Ureibacillus*
SV-5576	0.8	0.63	0.017	14	6.80E-03	2.10E-03	0	Bacteroidetes	*Natronoflexus*
SV-162	0.79	0.57	0.033	17	1.40E-01	5.70E-02	0	Proteobacteria	*Sphingopyxis*
SV-64	0.78	0.68	0.013	14	9.10E-02	4.40E-02	1.10E-03	Bacteroidetes	*Algoriphagus*
SV-1974	0.77	0.83	0.003	16	1.10E-02	2.10E-02	0	Firmicutes	*Symbiobacterium*
SV-630	0.76		NA		4.50E-03	1.30E-02	0	Proteobacteria	*Brucella*
SV-9181	0.76		NA		2.60E-03	1.70E-03	0	Firmicutes	*Caldalkalibacillus*
SV-19508	0.76		NA		1.10E-03	0	0	Proteobacteria	*Bdellovibrio*
SV-6670	0.75		NA		8.70E-03	4.70E-04	0	Proteobacteria	*Salinispirillum*
SV-10781	0.74		NA		2.60E-03	1.20E-03	0	Bacteroidetes	*Flavobacterium*
SV-658	0.74		NS		2.70E-02	1.90E-02	0	Verrucomicrobia	*Luteolibacter*
SV-2107	0.74	0.69	0.013	13	2.70E-02	9.80E-03	0	Planctomycetes	*Pirellula*
SV-998	0.73	0.54	0.035	15	1.10E-01	2.50E-02	0	Planctomycetes	*Thermostilla*
SV-5435	0.73		NA		2.40E-03	5.30E-03	0	Firmicutes	*Ruminiclostridium*
SV-1545	0.73		NS		1.90E-02	1.60E-02	0	Verrucomicrobia	*Luteolibacter*
SV-1141	0.72		NA		3.10E-02	6.60E-03	0	Bacteroidetes	*Pedobacter*
SV-3861	0.72		NA		4.40E-03	2.00E-03	0	Bacteroidetes	*Leadbetterella*
SV-2082	0.72	0.55	0.35	14	1.40E-02	1.50E-02	0	Proteobacteria	*Pseudorhodoplanes*
SV-166	0.71		NA		1.20E-02	9.40E-02	0	Proteobacteria	*Rheinheimera*
SV-1210	0.71		NA		5.50E-02	3.20E-02	0	Verrucomicrobia	*Diplosphaera*
SV-1745	0.71		NS		4.30E-02	4.60E-03	0	Bacteroidetes	*Flaviaesturariibacter*
SV-2869	0.71	0.64	0.017	15	2.10E-02	9.10E-03	0	Actinobacteria	*Thermobispora*
SV-1266	0.7		NS		3.70E-02	1.90E-02	0	Proteobacteria	*Steroidobacter*
SV-693	0.7	0.59	0.025	17	3.40E-02	5.90E-02	0	Actinobacteria	*Thermopolyspora*

NS, nonsignificant with a *P*-value larger than 0.05.

NA, not available; SV occurred in <11 composts.

a Point biserial correlation coefficient.

b Spearman correlation of the relative abundance of an SV with mean growth promotion including all composts in which the SV was present; with Benjamini–Hochberg adjusted *P*-value.

c Highly suppressive composts (H, I, K, L and Q).

d Rhizoplanes of plants grown in highly suppressive compost substrates inoculated with *P. ultimum*.

To further corroborate the relevance of the 75 SVs, relative abundance of each SV was correlated with mean disease suppression using all 17 composts, i.e. including those with an intermediate disease suppression (composts C, F, G, J, M, O and P). Correlations were calculated for the 40 SVs that occurred in at least 11 composts to ensure the inclusion of more composts than the five least and the five most suppressive ones. Of them 33 SVs showed a significant positive correlation (rho > 0.5 and *P*-value < 0.05) with mean disease suppression (Table S11, Supporting Information), reinforcing their potential involvement in disease suppression or indication of suppressive composts. Twelve of the significantly positively correlated SVs were identified to genus-level including *Sphingopyxis*, for which suppression has been suggested in the literature (Table 1; Table S11, Supporting Information).

## DISCUSSION

Compost bacteria likely play an important role in the activities of growth promotion and disease suppression of compost applications. Therefore, growth promotion and disease suppression of 17 composts were assessed with a bioassay including cress plants and the pathogen *P. ultimum*. Analyses revealed that both traits were related to physico-chemical compost characteristics and bacterial communities in composts as well as in the rhizoplanes of plants grown in compost substrates. At the community level, bacterial community structures were significantly correlated with growth promotion and disease suppression. Finally, 28 and 75 SVs, which were strongly associated with the five most growth-promoting and the five most disease-suppressive composts, were identified. Their abundances were tracked in the composts and the rhizoplanes and their taxonomies were determined and compared with the literature in order to evaluate their potential roles or indicative functions in both activities.

### Bacterial communities in composts and rhizoplanes

The 17 composts investigated in this study represented a range of green waste composts from commercial composting facilities, with composts differing in starting material and maturity. This diversity is reflected in differences of physico-chemical parameters (Tables S1 and S2, Supporting Information). Metabarcoding of bacterial communities showed that the composts contained distinct and specific bacterial community structures, which differed strongly from bacterial communities in the control matrix, which was a standard peat substrate. Only relatively small numbers of SVs (1.2%) were shared among all composts (Table S7, Supporting Information). Variability between replicates of the same compost was remarkably small, despite the heterogeneous structure of composts. The NMDS ordination of bacterial community structures revealed a cluster of composts with manure and mature compost among their starting materials, indicating the impact of these materials on bacterial communities in the final compost products. However, statistical support based on a balanced selection of composts is needed to test for the impact of starting materials on bacterial community structures. In another study using metabarcoding of a ribosomal marker, specific bacterial community structures in composts have been reported as well, and were related to age, starting materials and preparation methods (Neher *et al*. 2013). They found that bacterial community structures differed significantly among three types of compost, of which all included ensilaged manure, and one of them hay and one hardwood in addition. In comparison to the present study, the starting materials of the three compost types were relatively similar, nonetheless bacterial communities were strongly affected by the starting material. In contrast, a metabarcoding study including 116 composts from 16 composting companies across China has not revealed significant differences of bacterial communities among composts based on different starting materials or different composting processes, but showed that pH, moisture and total nitrogen of the composts affected bacterial community structures to some degree (Wang *et al*. 2020).

In the present study, bacterial communities in rhizoplanes of plants grown in compost substrates were strongly influenced by the compost applications. Similarly, bacterial communities, which were assessed using terminated restriction fragment length polymorphism, in the rhizosphere of tomato plants grown in the field differed according to the organic amendment, i.e. three different compost manure-based and two different plant-based amendments, which the plants had received during seedling production in the green house (Jack *et al*. 2011). Furthermore, the effect of the different amendments decreased over time. Bacterial communities in cress rhizoplanes included about three times (3.2) more bacterial SVs from the compost than from the control matrix, although compost substrates were composed of a mixture of compost and control matrix at a ratio of 20:80. This may indicate that that compost bacteria are better adapted to the nutrient-rich rhizoplanes and outcompete bacteria derived from the control matrix or that plants select and shape their closely associated microbiota. Evidence for interactions between plants and their root microbiome, e.g. via plant exudates consumed by microorganisms, has been reviewed by Sasse, Martinoia and Northen (2018). In the present study, the selectivity of the rhizoplane was further supported by the less distinct rhizoplane bacterial community structures among composts as compared with the large differences of compost bacterial community structures among composts. In contradiction to our initial hypothesis, suppression and growth promotion were related to a slightly smaller extent to bacterial communities in the rhizoplanes grown in compost substrates than to those in the composts themselves. Therefore, the search for indicative SVs was based on bacteria in composts as opposed to those in the rhizoplanes.

### Growth promotion

Of the 17 composts, nine showed a significant growth promotion. A weak positive correlation of growth promotion and nitrate content in composts was observed, suggesting at least partially a nutrient based growth promotion by the directly plant-available nitrate (e.g. Stitt 1999). Growth promotion was also significantly correlated with a change in bacterial community structures. Similarly, other studies revealed correlations of growth promotion with measures of microbial activity such as soil respiration and enzyme activity after compost applications (Bonanomi *et al*. 2014; Pane *et al*. 2015). Because of the correlation of growth promotion and nitrate and the similar amounts of variances both factors explained in bacterial community structures, both factors may be important bacterial community structures. Growth promotion due to compost application has also been related to the presence of growth-promoting bacteria (De Brito, Gagne and Antoun 1995). In the present study, indicator analysis identified 28 SVs that were potentially involved in or indicating growth promotion. For six of these genera, i.e. *Acinetobacter*, *Bacillus*, *Bradyrhizobium*, *Brevundimonas*, *Microvirga* and *Streptomyces*, a role in growth promotion has previously been reported (Table S11, Supporting Information). The most strongly associated of them was *Microvirga* and the most abundant was *Bradyrhizobium*. Both genera are known for symbiotic nitrogen fixation in root nodules with legumes (Ardley *et al*. 2012) but it has been shown that at least *Bradyrhizobium* promoted growth also in non-legumes, such as radish (Antoun *et al*. 1998) and rice (Chaintreuil *et al*. 2000) possibly due to nitrogen fixation of endophytic strains without the requirement of specialized plant organs (Bhattacharjee, Singh and Mukhopadhyay 2008). However, the importance of *Bradyrhizobium* in growth promotion in our system is less likely, because the *Bradyrhizobium*-SV was more abundant in the control matrix than in the most growth-promoting composts. Beside nitrogen fixation, growth promotion may have resulted from the production of different types of siderophores, for example by *Acinetobacter*, with their ability to provide nutrients to the plants by solubilizing phosphates and zinc oxides (Rokhbakhsh-Zamin *et al*. 2011). The production of phytohormones leading to increased growth has been shown for *Bacillus* (reviewed in Santoyo, Orozco-Mosqueda and Govindappa 2012) and *Streptomyces* strains (reviewed in Olanrewaju and Babalola 2019), while strains of the latter have also been related to the production of siderophores and fixation of nitrogen. Growth promotion as well as stress tolerance of rice to arsenic has been shown to be mediated by a *Brevundimonas diminuta* strain (Singh *et al*. 2016). The abovementioned six genera may have contributed to growth promotion of cress plants; however, there were three genera, i.e. *Hyphomicrobium* sp., *Peredibacter* sp. and *Panacagrimonas* sp., for which growth promotion has not yet been reported and 19 SVs whose genera have not yet been described. Finally, further experiments will be required to explore whether these taxa are directly involved in growth promotion or whether they represented indicators for growth-promoting compost properties.

### Disease suppression

Growth promotion and disease suppression were not correlated, suggesting different underlying mechanisms. Similarly, the comparison of disease suppression against *R. solani* and growth promotion of *Lactuca sativa* after the application of 14 different organic amendments at different stages of decomposition showed that organic amendments had different activities in both traits and their extent changed with decomposition time, which revealed a trade-off between the traits (Bonanomi *et al*. 2020). Furthermore, total nitrogen content, carbon-to-nitrogen ratio and specific compounds classified according to functional carbon groups explained disease suppression and growth promotion only to a limited extent.

In the present study, disease suppression in the *Pythium*-cress system was negatively correlated with compost age, nitrate content and total inorganic nitrogen content. The latter three compost characteristics were positively correlated with each other (rho > 0.6, *P* < 0.05), which is known for composts (Grebus, Watson and Hoitink 1994). Age, nitrate content and disease suppression explained significant portions of bacterial community structures (9.5–17.6%). Compost age, which ranged from 35 to 320 days, was considered a proxy for compost maturity. In a review focusing on the effect of organic material degraded to different degrees (representing age) on suppression of various pathogens, Bonanomi *et al*. (2010) concluded that it is difficult to relate compost maturity to suppression across studies due to complex interactions of starting materials, composting processes and maturity measures and due to the lack of a generally applicable definition of maturity. For example, Grebus, Watson and Hoitink (1994) found that composts from yard trimmings became suppressive against *Pythium* already 6 d after starting the composting process, and remained suppressive until the end of the study after a short (10 d) curing phase. In contrast, Chen, Hoitink and Schmitthenner (1987) reported that a bark compost was only suppressive after the thermal phase. Stone, Traina and Hoitink (2001) found that suppression was lost after an extended curing phase of over one year. In the present study, young composts (35–106 d) with relatively short curing time and low nitrogen content were most suppressive. This was regardless of the different starting materials, which included plant residues only or mature compost and manure in addition, and regardless of composting facilities, i.e. the five most suppressive composts were obtained from four different providers (Table S1, Supporting Information).

Microbial communities play an important role in suppression of composts against *Pythium*, which can be inferred from the significant correlation of disease suppression and bacterial community structures in the present study. This is supported by a loss of suppression after sterilization (Boehm and Hoitink 1992; Craft and Nelson 1996) and the correlation of suppression with general microbiological measures (Chen, Hoitink and Madden 1988; Scheuerell, Sullivan and Mahaffee 2005). Furthermore, a comparison of microbial communities of nine composts with different degrees of disease suppression against *Pythium* revealed the presence of the acidobacterial subgroup Gp14 and the fungal class Cystobasidiomycetes only in suppressive compost–peat mixtures, and a positive correlation of the bacterial phylum Actinobacteria with suppression (Yu *et al*. 2015). In the present study, the search for indicator species revealed 75 relatively low-abundant (≤0.11%) SVs, which were strongly associated with disease suppression. The 75 low abundant SVs strongly associated with disease suppression may suggest that combinations of several SVs, i.e. bacterial consortia, are involved in or are indicative of developing disease suppression. Among the most strongly associated SVs in the present study were three members of the genus *Ureibacillus*. The genus *Ureibacillus* includes isolates obtained from composts (Weon *et al*. 2007), but for which suppressive activities have not yet been reported. Five of the strongly associated SVs that were also identified to genus level, i.e. *Sphingopyxis*, *Steroidobacter*, *Flavobacterium*, *Rheinheimera* and *Pedobacter* have been reported in the context of disease suppression against oomycetal or fungal pathogens. The *Sphingopyxis*-SV, besides being strongly associated with highly suppressive composts, was also positively correlated with suppression in all 17 composts, supporting its potential involvement in suppression. Members of the genus *Sphingopyxis* have been found in disease suppressive soils against *Fusarium oxysporum* f. sp. *radicis*-*cucumerinum* (Klein *et al*. 2013) using 16S pyrosequencing. Furthermore, a consortium of seven bacterial strains including two *Sphingopyxis* strains was able to suppress *Fusarium oxysporum* in a hydroponic system, although each individual strain was not effective (Fujiwara *et al*. 2016). Strains of *Flavobacterium* have already been successfully used as biocontrol agents. For instance, *Flavobacterium balustinum* strain 299, which has been applied with composted bark, appeared to increase suppression against wilt caused by *Rhizoctonia solani* in radish (Kwok *et al*. 1987), and *Flavobacterium johnsoniae* strain GSE09 suppressed *Phytophthora capsici* on pepper (Sang and Kim 2012). Disease suppression of *Steroidobacter* and *Pedobacter* has been indicated by correlations to suppression or *in vitro* tests. For example, after the amendment of almond shell composts in an avocado orchard, OTUs of the genus *Steroidobacter* have been correlated to soil suppressive against white root rot in avocado caused by the fungus *Rosellinia necatrix* (Vida *et al*. 2016). In other studies, the application of biochar has been related to the presence of *Steroidobacter* in the rhizosphere of pepper plants protected against *Phytophthora* (Wang *et al*. 2019) and in the rhizosphere of Tobacco plants protected against *Ralstonia solanacearum* (Zhang *et al*. 2017). OTUs of *Pedobacter* were associated with the rhizosphere of wheat plants growing within bare patches of a wheat field due to infection with *Rhizoctonia solani* and a corresponding *Pedobacter*-isolate showed fungal growth inhibition *in vitro* (Yin *et al*. 2013). A different *Pedobacter* isolate produced a chitinase with antifungal activity *in vitro* (Song, Seo and Jung 2017). Another genus that has shown potential for disease suppression due to the ability to produce antimicrobial substances was *Rheinheimera* (Kalinovskaya, Romanenko and Kalinovsky 2017).

In summary, the 75 SV strongly associated with disease suppression included five genera with known potential for disease suppression against fungal and oomycetal pathogens, but not specifically against *Pythium* spp., and many more novel and potentially suppressive taxa were detected. Among the most interesting SVs were the genus *Ureibacillus* for which disease suppression has not been described and *Sphingopyxis* for which such a trait has already been shown. Different SVs associated with highly growth-promoting composts and with highly suppressive composts, suggesting that these activities rely on different mechanisms. In order to elucidate their roles, these SVs need to be isolated and subjected to functional tests. The combination of metagenomics, isolation and functional prioritization followed by functional genomic characterization of isolated strains holds the potential to shed more light on compost microbiota responsible for disease suppression (Lutz *et al*. 2020), which builds the centerpiece in developing sustainable biocontrol strategies. Furthermore, activity in disease suppression may also be attributed to additional groups of microorganisms such as fungi and protists. Therefore, an important question for future studies is which specific groups of all microbiota play a role in plant disease suppression.

### Towards prediction of compost-associated disease suppression

Development of reliable and easy-to-use diagnostic tools is highly desirable for a more efficient production and use of composts for combating yield losses caused by soil-borne diseases. An effort towards predicting disease suppression of composts against various pathogens has been made before (Chen, Hoitink and Madden 1988; Mehta *et al*. 2018). For example, microbial activity determined as hydrolysis of fluorescein diacetate and biomass measured as extractable phospholipid phosphate content have been proposed as prediction tools for suppression against damping-off caused by *P. ultimum* (Chen, Hoitink and Madden 1988); however, contradicting results, in which fluorescein diacetate hydrolysis was not related to suppression of *P. ultimum*, have questioned these results later on (Pane *et al*. 2011). Mehta *et al*. (2018) have developed a PCR-based tool for screening suppression of composts against *Fusarium* based on the presence of a specific 16S rRNA gene fragment, which was determined using DGGE community profiling, indicating the potential of molecular marker-based diagnostic tools. In the present study, we focused on bacterial communities in composts as compared with those in the rhizoplanes. This has the great advantage of predicting growth promotion and disease suppression without time-consuming cultivation of plants and extraction of rhizoplanes, and, in the future, to develop diagnostic tools based on the analysis of compost samples. Prediction of growth promotion and disease suppression could be based on the detection of the 28 and 75 associated SVs, respectively, regardless of whether they are responsible for or indicative of these traits. For that, a large number of composts with different degrees of growth promotion and disease suppression is required for validation of marker-SVs. Furthermore, assays for quantification of markers, such as qPCR, may have to be developed based on lists of SVs for a more cost-efficient predictive analysis.

## CONCLUSIONS

The 17 composts differed in their abilities to promote cress plant growth and to suppress the disease symptoms caused by *P. ultimum*. Lack of correlation between the two studied traits, i.e. cress plant growth promotion and disease suppression against*P. ultimum*, implied different underlying mechanisms, and therefore have to be evaluated independently. Composts included distinct and diverse bacterial communities and compost applications led to an enrichment of compost bacteria in the rhizoplanes grown in compost substrates. On average, 3.2 times more bacteria originated from the compost than from the matrix, although the growth substrates included four times more matrix than compost. Both traits correlated significantly with compost bacterial communities, supporting the importance of bacteria for these traits. Growth promotion was significantly correlated with nitrate content and both affected bacterial community structures to a similar degree revealing complex interdependencies among them. Twenty-eight SVs were strongly associated with growth promotion, of which the six genera *Acinetobacter*, *Bacillus*, *Bradyrhizobium*, *Brevundimonas*, *Microvirga* and *Streptomyces* have been described for this trait. Disease suppression of *P. ultimum* correlated negatively with compost age and nitrate content. Young composts were most suppressive against *P. ultimum* with a relatively short curing time. Among the 75 SVs that were associated with highly suppressive composts, the genera *Ureibacillus* and *Sphingopyxis* represented promising candidates for disease suppression. SVs associated with plant growth promotion and disease suppression need to be validated and assessed whether they are indicative of or directly involved in the two traits. Selected SVs represent a basis for the development of diagnostic tools to predict growth promotion and disease suppression against *P. ultimum*, which is highly desirable for targeted compost production and application for agricultural use.

## ACKNOWLEDGEMENTS

We are grateful to all compost producers who provided us with the large number of composts and to Tabea Koch, Urs Büchler and Dominic Spichtig for their valuable support in the lab.

## Supplementary Material

fiab134_Supplemental_File
