# Supplementary material for: Indicative bacterial communities and taxa of disease-suppressing and growth-promoting composts and their associations to the rhizoplane
Source: FEMS Microbiol Ecol. 2021 Sep 21;97(10):fiab134. doi: 10.1093/femsec/fiab134 (PMC8478479; doi:10.1093/femsec/fiab134)
Supplement: fiab134_Supplemental_File [file fiab134_supplemental_file.pdf]

Supplementary information

Supplementary Figure 1: Experimental design of the bioassay. 2

Supplementary Figure 2: Correlations of mean percent growth promotion and mean compost characteristics 3

Supplementary Figure 3: Disease suppression among compost substrates 4

Supplementary Figure 4: Correlation plots of mean percent disease suppression and compost characteristics 5

Supplementary Figure 5: Partitioning of robustly detected SVs among substrates and rhizoplanes. 6

Supplementary Figure 6: Partitioning of robustly detected SVs among substrates and rhizoplanes of the most and the least growth promoting (A) and disease suppressing (B) composts. 7

Supplementary Table 1: Composition of starting materials 8

Supplementary Table 2: Compost characteristics of the 17 composts 9

Supplementary Table 3: Cress shoot weight (mean and standard deviation) and disease symptoms (median) 10

Supplementary Table 4: Spearman correlations of mean disease suppression or mean growth promotion with compost characteristics. 11

Supplementary Table 5: Sequences assigned to plant organelles 12

Supplementary Table 6: Differences of bacterial community structures among composts and the matrix 13

Supplementary Table 7: Number and percentage of 15,236 SVs occurring in increasing combinations from one to 17 different composts. 14

Supplementary Table 8: Effect of disease suppression, growth promotion and compost characteristics on bacterial community structures. 15

Supplementary Table 9: Partitioning of SVs among five most and least active composts. 16

Supplementary Table 10: Relative abundance and taxonomy of SVs associated with growth promoting composts 17

Supplementary Table 11: Relative abundance, occurrence and taxonomy of 75 SVs associated to strongly suppressive composts 18

Supplementary References 20

Supplementary Figure 1: Experimental design of the bioassay. Two batches were tested with 9 composts each where compost H was included in both batches. Three replicates for metabarcoding (red circles) and three replicates of cress biomass (green triangles) were sampled. In total, 174 samples were obtained for metabarcoding including 54 compost samples (2 batches x 9 composts x 3 replicates), 3 matrix samples, 108 rhizoplane samples of cress grown in compost substrate (2 batches x 2 inoculation levels x 9 composts x 3 replicates) and 12 rhizoplane samples of cress grown in control matrix (2 batches x 2 inoculation levels x 3 replicates). In total, 240 cress biomass samples were obtained including 182 samples from plants grown in compost substrates (2 batches x 9 composts x 4 inoculation levels x 3 replicates) and 24 samples from plants grown in the control matrix (2 batches x 4 inoculation levels x 3 replicates).

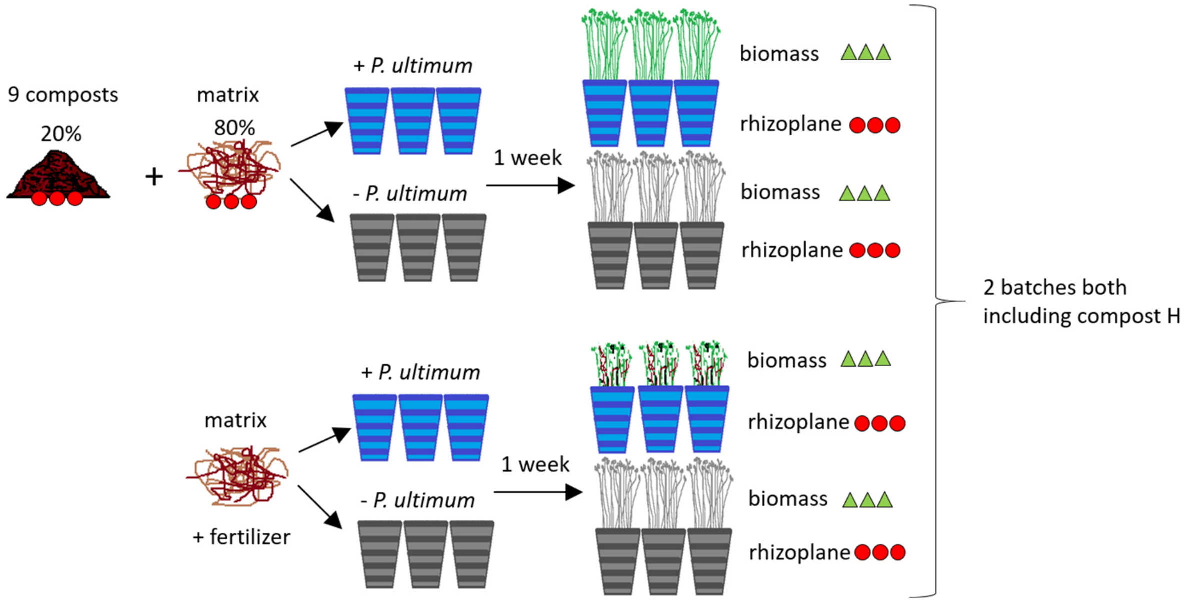

Supplementary Figure 2: Correlations of mean percent growth promotion and mean compost characteristics such as nitrate [mg N (kg dry weight)$^{-1}$], inorganic nitrogen [mg N (kg dry weight)$^{-1}$], salinity [g KCleq (kg dry weight)$^{-1}$], age [d], ammonia [mg N (kg dry weight)$^{-1}$], dry matter [%], content of soluble humic substances (OD 550) and pH. Plots were ordered according to the absolute value of the strength of the Spearman correlation (rho) and a linear trendline was added only for significant correlations ($p < 0.05$). Correlations were based on 17 composts, except for age only 12 were available. Statistics of correlations are also presented in Supplementary Table 4. Mean growth promotion was defined as the percent increase in shoot weight of plants that were grown in compost substrates compared to the control matrix both without inoculation with the pathogen.

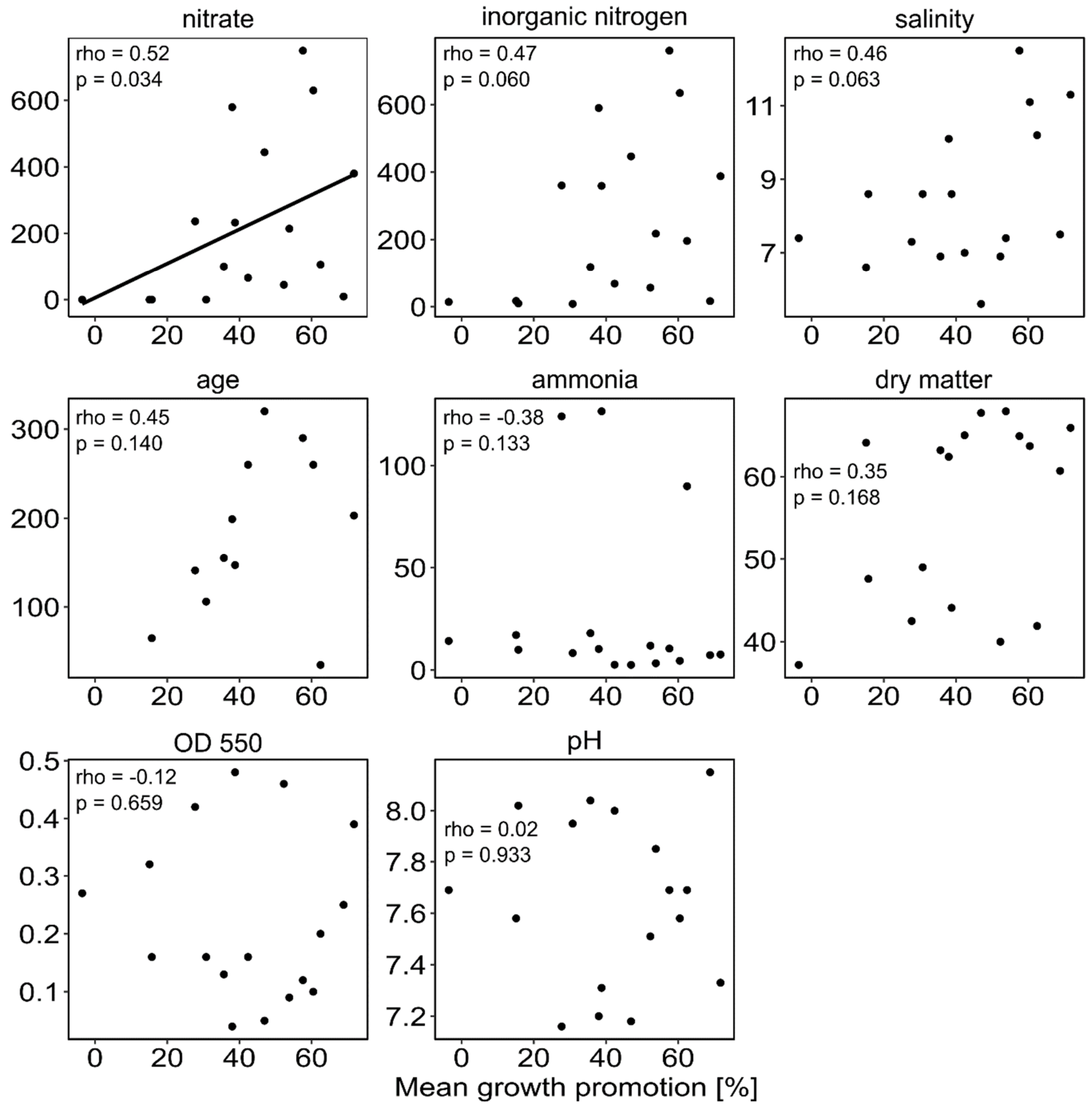

Supplementary Figure 3: Disease suppression among compost substrates (A-Q) and the control matrix (m) inoculated with either 0.25, 0.5 or 1 g of *P. ultimum*-millet-mix (L of substrate)$^{-1}$ of the first (A) and the second (B) batch of composts. Percent disease suppression was defined as the relative shoot weight of *P. ultimum*-inoculated and uninoculated cress plants for each compost treatment and the control matrix. Letters show significant differences among substrates (TukeyHSD-tests, $p < 0.05$). ANOVA was performed separately for each experiment and inoculation-level and overall statistics for 0.25, 0.5 and 1 g L^{-1} were $F = 1.2$ and $p = 0.358$, $F = 2.6$ and $p = 0.038$ as well as $F = 19.8$ and $p = 4 \times 10^{-8}$ in batch one (A) and $F = 3.0$ and $p = 0.019$, $F = 7.2$ and $p = 0.0001$ as well as $F = 8.0$ and $p = 6 \times 10^{-5}$ in batch 2 (B). Compost H was tested in both experiments.

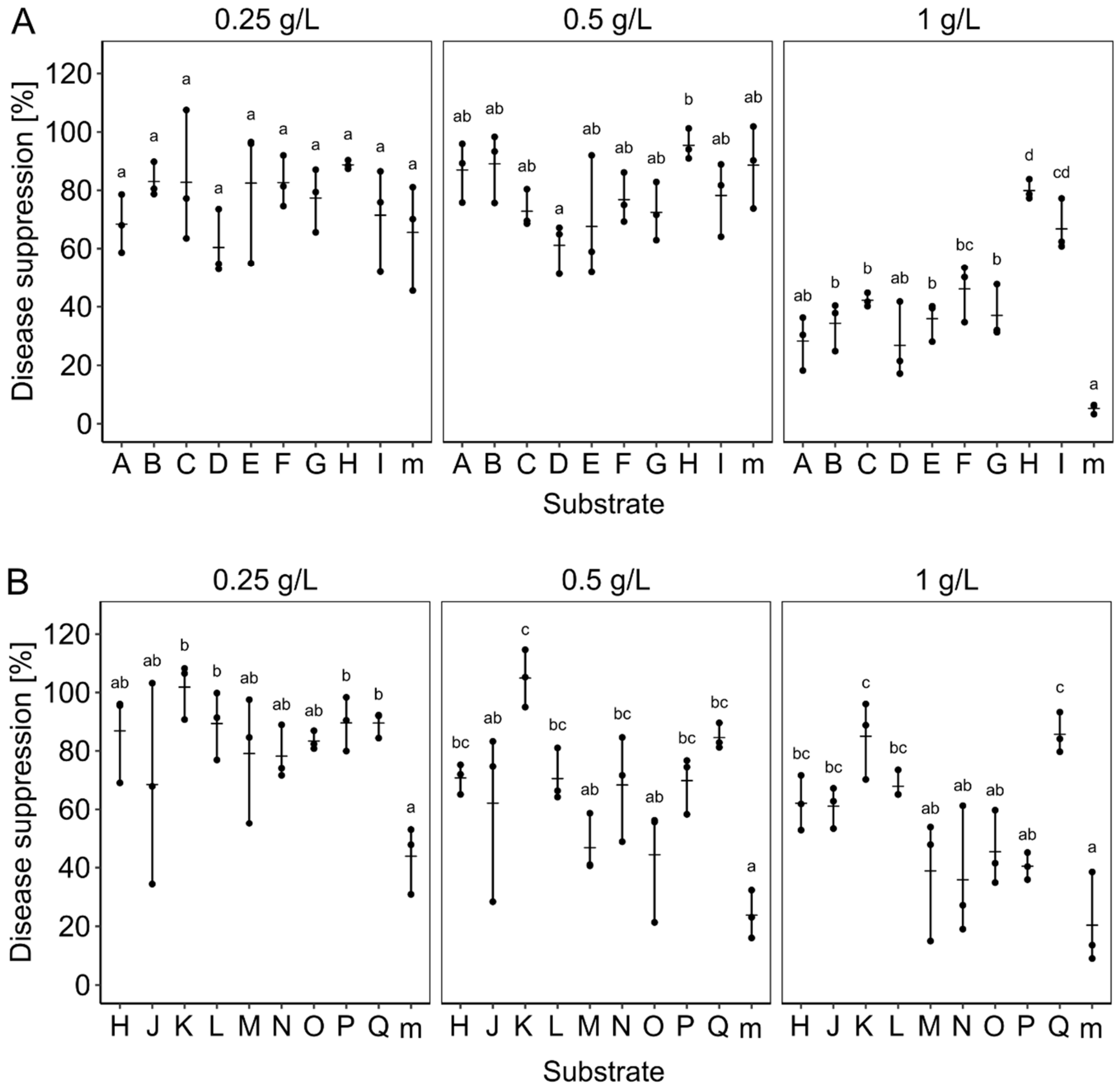

Supplementary Figure 4: Correlation plots of mean percent disease suppression and compost characteristics such as age [d], nitrate [mg N (kg dry weight)$^{-1}$], inorganic nitrogen [mg N (kg dry weight)$^{-1}$], dry matter [%], content of soluble humic substances (OD 550), ammonia [mg N (kg dry weight)$^{-1}$], pH and salinity [g KCleq (kg dry weight)$^{-1}$]. Plots were ordered according to the absolute value of the strength of the Spearman correlation (rho) and a linear trendline was added only for significant results ($p < 0.05$). Correlations were based on 17 composts, except for age only 12 were available. Statistics of correlations are also presented in Supplementary Table 4. Percent disease suppression was defined as the relative shoot weight of *P. ultimum*-inoculated and uninoculated cress plants for each compost treatment.

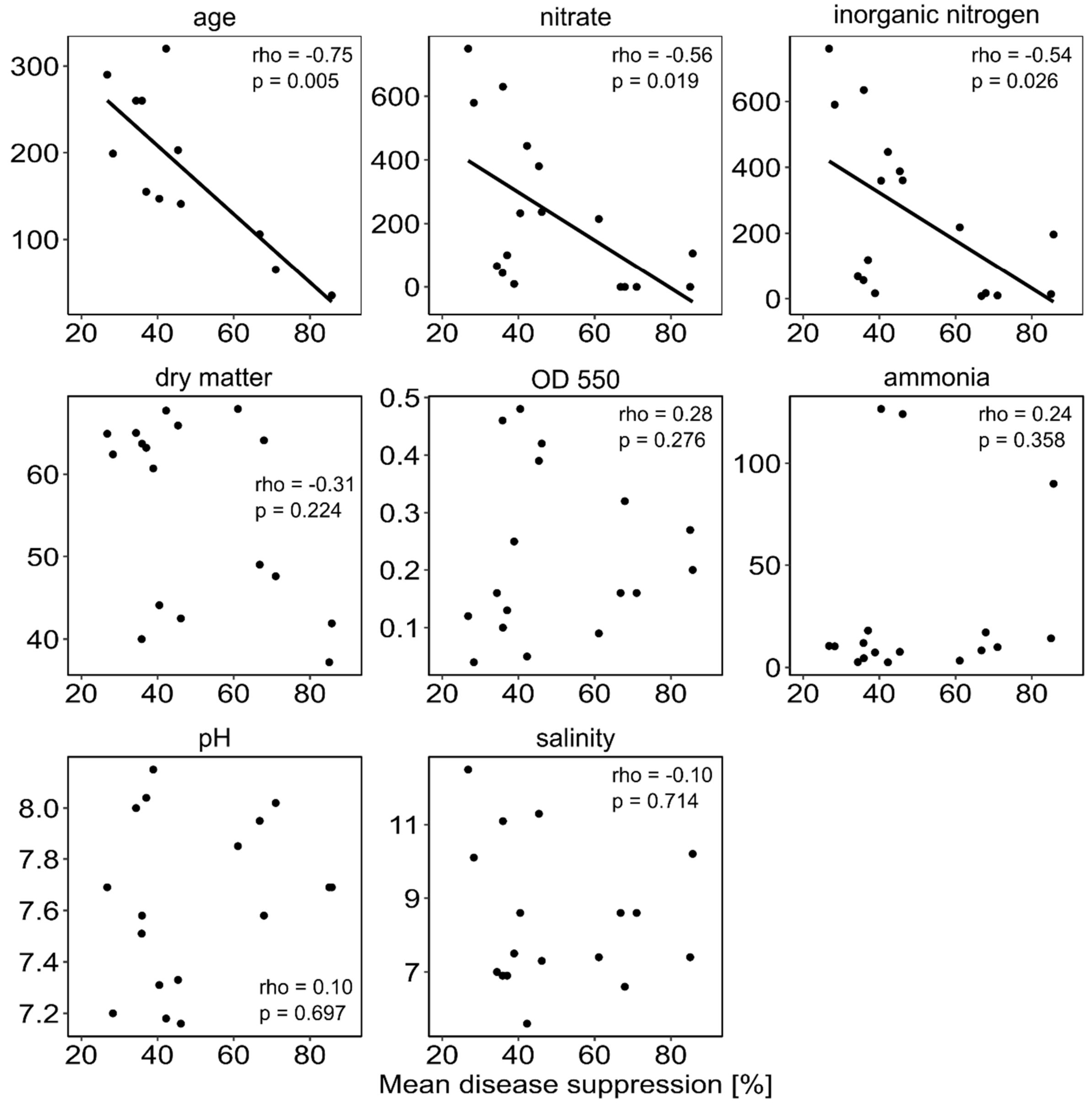

Supplementary Figure 5: Partitioning of robustly detected SVs among substrates and rhizoplanes. (A) Venn diagram displaying the occurrence of SVs in each compartment, i.e., compost (brown), matrix (black), rhizoplanes of cress plants grown in compost substrate (“rhizo c”, pink dashed line) and rhizoplanes of cress plants grown in the matrix (“rhizo m”, blue dashed line). Rhizoplane data derived from plants inoculated with *P. ultimum* are presented. The numbers outside of the circles of the Venn diagram show the number of SVs and the mean percentage of SVs in each compartment. The numbers in the different areas of the Venn diagram correspond to mean percentages of SVs from the 17 composts, the matrix, the corresponding rhizoplanes and their intersections. Standard deviations are presented in parenthesis. Data were obtained using a subsampling procedure to 18,743 sequences with 100 iterations. (B) Partitioning of SVs in rhizoplanes of plants grown in compost substrates (“rhizo c”). Percentages correspond to SVs that were also found in compost (“c”), in compost and the matrix (“c+m”), the matrix (“m”) and in rhizoplanes of plants grown in the control matrix (“rhizo m”), or that were only detected in rhizoplanes of plants grown in the compost substrates (“rhizo c”).

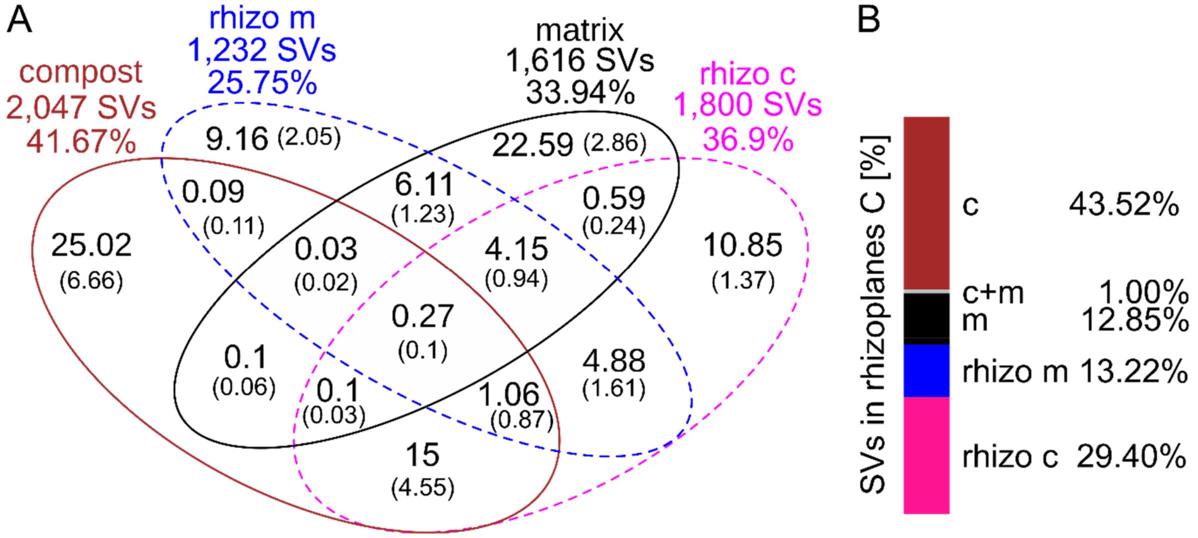

Supplementary Figure 6: Partitioning of robustly detected SVs among substrates and rhizoplanes of the most and the least growth promoting (A) and disease suppressing (B) composts. The numbers in the different areas of the Venn diagram correspond to mean percentages of SVs. The first number is the mean percentage of SVs in the 5 most active and the second number the 5 least active compost for growth promotion (A) and disease suppression (B). Colours represent different compartments, i.e., compost (brown), matrix (black), rhizoplanes of cress plants grown in compost substrate ("rhizopl. compost", pink dashed line) and rhizoplanes of cress plants grown in the matrix ("rhizopl matrix", blue dashed line). Rhizoplane data derived from uninoculated plants (A) and plants inoculated with *P. ultimum* (B) are presented. Data were obtained using a subsampling procedure to 18,743 (A) and 13,173 (B) sequences with 100 iterations. The Asterisk represents a significant difference of percentage of SVs of the five most active and least active composts ($p < 0.05$). Statistics of all Student t-tests are presented in Supplementary Table 9.

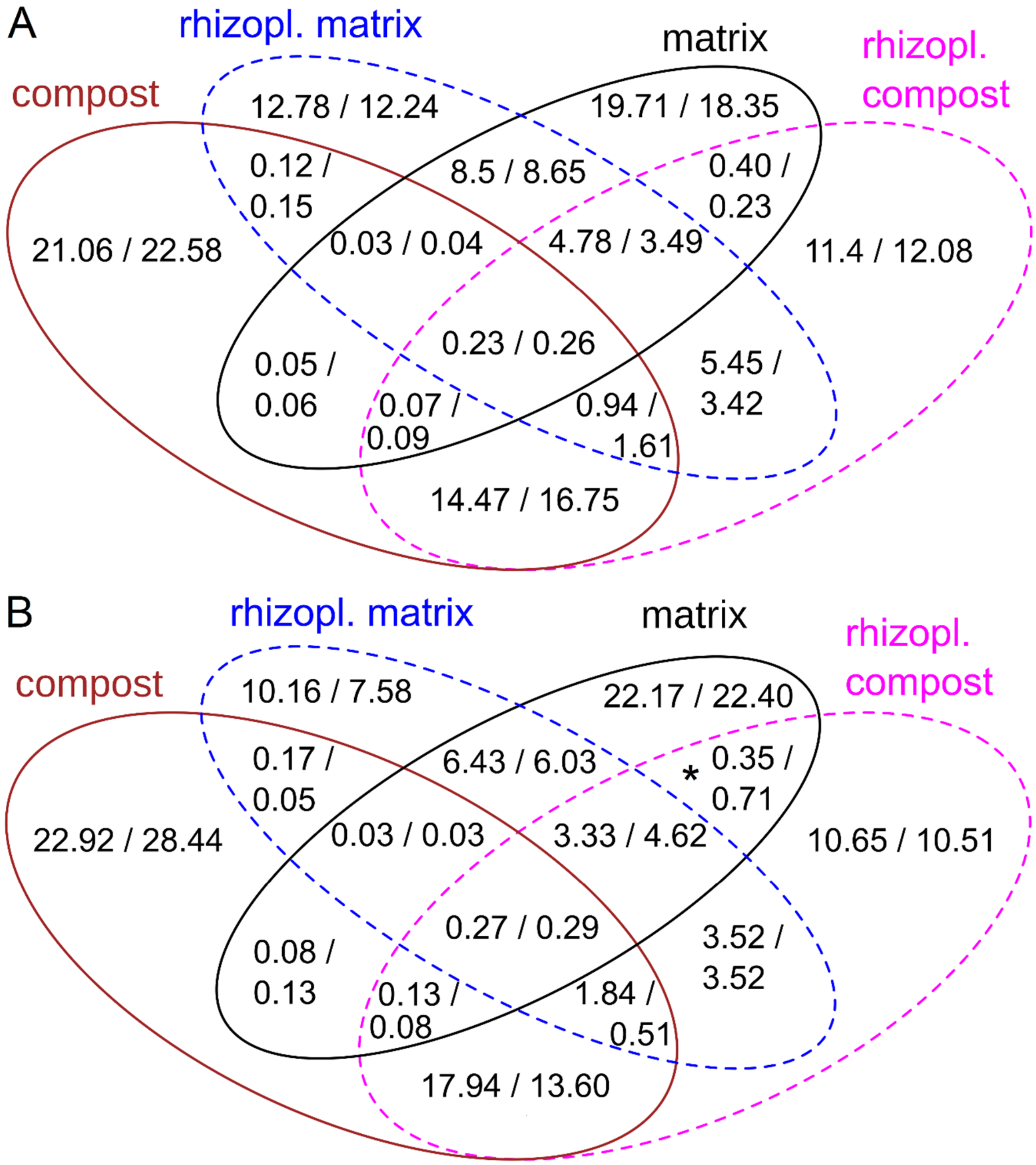

Supplementary Table 1: Composition of starting materials in percent used in the composting process of each of the 17 composts obtained from different commercial compost providers.

Compost	Provider	Plant residues[a]	Soil / humus	Fibre chalk[b]	Mature compost	Manure[c]	Wet biomass	Biochar and/or additives
A	A	60	10	0	10	15	0	5 (biochar, leonardit, biolit, betonit)
B	A	40	8	0	8	33	0	12 (biochar, biolit)
C	A	58	8	0	8	25	0	1 (biolit)
D	A	40	8	0	8	40	0	5 (biochar, biolit)
E	A	45	8	0	10	33	0	5 (biochar, biolit)
F	B	90	0	10	0	0	0	0
G	C	75	10	0	10	5	0	0
H	C	80	10	0	10	0	0	0
I	C	75	10	0	10	5	0	0
J	D	NA	NA	NA	NA	NA	NA	NA
K	D	NA	NA	NA	NA	NA	NA	NA
L	E	NA	NA	NA	NA	NA	NA	NA
M	E	NA	NA	NA	NA	NA	NA	NA
N	B	NA	NA	NA	NA	NA	NA	NA
O	B	100	0	0	0	0	0	0
P	B	95	2	0	0	0	0	3 (diatomaceou s earth)
Q	B	70	0	0	0	0	30	0

a plant residues included leaves, twigs, roots, green waste, grass and hay
b fibre chalk from a paper production company
c manure from cow, horse, pig, chicken
NA not available

Supplementary Table 2: Compost characteristics of the 17 composts that were used to define different qualities such as age, dry matter, pH, salinity, content of soluble humic substances of the extract (OD 550), total inorganic nitrogen, nitrate and ammonia.

Compost	Age [d]	Dry matter content [%]	pH	Salinity [g KCleq[1] (kg dry weight)$^{-1}$]	OD 550 [nm]	Nmin[2] [mg N (kg dry weight)$^{-1}$]	NO_3^- [mg N (kg dry weight)$^{-1}$]	NH_4^+ [mg N (kg dry weight)$^{-1}$]
A	199	62.4	7.2	10.1	0.04	589.9	579.5	10.3
B	260	65.0	8.0	7.0	0.16	68.3	65.7	2.6
C	320	67.7	7.2	5.6	0.05	446.5	444	2.5
D	290	64.9	7.2	12.5	0.12	759.9	749.4	10.5
E	260	63.7	7.6	11.1	0.10	634.4	629.9	4.5
F	141	42.5	7.2	7.3	0.42	360.5	236.4	124.1
G	155	63.2	8.0	6.9	0.13	118.3	100.3	18
H	65	47.6	8.0	8.6	0.16	9.9	0	9.9
I	106	49.0	8.0	8.6	0.16	8.3	0	8.3
J	NA	67.9	7.9	7.4	0.09	217.7	214.4	3.3
K	NA	37.2	7.7	7.4	0.27	14.2	0	14.2
L	NA	64.1	7.6	6.6	0.32	17.1	0	17.1
M	NA	60.7	8.2	7.5	0.25	16.6	9.3	7.3
N	NA	40.0	7.5	6.9	0.46	56.5	44.6	11.9
O	203	65.9	7.3	11.3	0.39	387.9	380.3	7.6
P	147	44.1	7.3	8.6	0.48	359.2	232.6	126.6
Q	35	41.9	7.7	10.2	0.20	196.1	106.1	90

1 potassium chloride equivalent
2 inorganic nitrogen, sum of ammonia and nitrate
NA not available

Supplementary Table 3: Cress shoot weight (mean and standard deviation) and disease symptoms (median) of plants grown in compost substrate (A-Q) or the control matrix (m). Bioassays were conducted in two batches including nine compost substrates each. Compost A-I were tested in the first batch, compost J-Q were tested in the second batch and compost H (H1 and H2) and the control matrix (m1 and m2) were included in both batches. Plants were inoculated with 1 g of *P. ultimum*-millet-mix (L substrate)$^{-1}$. The severity of disease symptoms of damping-off was classified in five categories according to the severity of disease, i.e., plants showing yellowing of leaves and growth reduction.

substrate	inoculation with *P. ultimum*	cress shoot weight [g]		disease symptoms[a]
		mean	SD	median
A	no	7.97	0.89	0
A	yes	2.25	0.73	2
B	no	8.22	0.54	0
B	yes	2.82	0.69	1
C	no	8.48	0.41	0
C	yes	3.58	0.20	1
D	no	9.10	0.51	0
D	yes	2.44	1.20	2
E	no	9.26	0.30	0
E	yes	3.33	0.63	1
F	no	7.37	0.34	0
F	yes	3.40	0.74	1
G	no	7.83	0.64	0
G	yes	2.90	0.73	1.5
H1	no	6.44	0.36	0
H1	yes	5.15	0.22	0
I	no	7.55	0.15	0
I	yes	5.04	0.68	0
m1	no	5.77	0.49	0
m1	yes	0.30	0.10	3.5
H2	no	5.82	0.53	0
H2	yes	3.61	0.55	1
J	no	7.47	1.96	0
J	yes	4.56	0.53	0.5
K	no	4.68	0.14	0
K	yes	3.98	0.62	0.5
L	no	5.59	0.19	0
L	yes	3.80	0.27	1
M	no	8.20	0.20	0
M	yes	3.19	1.71	1
N	no	7.40	0.07	0
N	yes	2.65	1.66	2
O	no	8.34	1.18	0
O	yes	3.78	1.07	1
P	no	6.74	0.21	0
P	yes	2.73	0.31	1
Q	no	7.89	0.31	0
Q	yes	6.76	0.55	0
m2	no	4.86	0.44	0
m2	yes	0.99	0.77	3

a Ordinal scale for severity of disease symptoms: 0 = no symptoms, 1 = few symptoms, 2 = medium symptoms, 3 = strong symptoms, 4 = no germination

Supplementary Table 4: Spearman correlations of mean disease suppression or mean growth promotion with compost characteristics of the 17 composts. Percent disease suppression was defined as the relative shoot weight of *P. ultimum*-inoculated and un-inoculated cress plants for each compost treatment. Mean growth promotion was defined as the percent increase in shoot weight of plants that were grown in compost substrates compared to the control matrix both without inoculation with the pathogen. Results from a correlation of mean disease suppression with mean growth promotion was also included.

Factor	Correlation with suppression			Correlation with growth promotion		
	rho	p-value	n	rho	p-value	n
age at arrival [d]	-0.75	0.005*	12	0.45	0.140	12
dry matter content [%]	-0.31	0.224	17	0.35	0.168	17
pH	0.10	0.697	17	0.02	0.933	17
Salinity [g KCleq[a] (kg dry weight)$^{-1}$]	-0.10	0.714	17	0.46	0.063	17
soluble humic substances (OD 550)	0.28	0.276	17	-0.12	0.659	17
nitrate [mg N (kg dry weight)$^{-1}$]	-0.56	0.019*	17	0.52	0.034*	17
ammonia [mg N (kg dry weight)$^{-1}$]	0.24	0.358	17	-0.38	0.133	17
Inorganic N [mg N (kg dry weight)$^{-1}$][b]	-0.54	0.026*	17	0.47	0.060	17
Growth promotion [%]	-0.34	0.178	17	NA	NA	NA

* p-value smaller than 0.05

a Potassium chloride equivalent

b sum of ammonia and nitrate

NA not available

Supplementary Table 5: Sequences assigned to plant organelles such as chloroplasts and mitochondria as well as to bacteria. Mean and standard deviation of numbers for each compartment including composts, control matrix and rhizoplanes of cress plants grown in either compost substrates or the untreated control matrix. Percentages of sequences assigned to chloroplast and mitochondria of all sequences. Plant organelle sequences were removed from the dataset for all following analyses.

compartment	Sequences assigned to									
	chloroplasts		mitochondria		bacteria		chloroplasts [%]		mitochondria [%]	
	mean	SD	mean	SD	mean	SD	mean	SD	mean	SD
compost	1.9	2.3	3.4	5.1	29,738.0	2,490.6	0.006	0.008	0.010	0.020
matrix	0.7	0.6	0	0	24,825.3	5,637.0	0.003	0.002	0	0
Rhizopl. of compost	1,071.1	1,413.5	394.4	442.4	28,862.4	4,879.3	3.489	4.487	1.290	1.420
Rhizopl. of matrix	2,792.9	5,175.8	1,015.4	1,764.9	27,543.9	5,633.9	7.946	13.737	2.910	4.690

Supplementary Table 6: Differences of bacterial community structures among composts and the matrix as well as rhizoplanes grown in different substrates, i.e., composts or matrix, with or without inoculation with the pathogen. Tests were performed using PERMANOVA and Bray Curtis dissimilarities of relative abundances. Compost and rhizoplane communities included three replicates for each treatment. Except, the rhizoplanes of plants grown in H and in the matrix, included six replicates which originated from two experimental batches.

	Pseudo-F	p-value
Compost communities	36.7	0.0001
Rhizoplanes communities inoculated with *P. ultimum*	6.3	0.0001
Rhizoplane communities not inoculated with *P. ultimum*	7.3	0.0001

Supplementary Table 7: Number and percentage of 15,236 SVs occurring in increasing combinations from one to 17 different composts. Only robustly detected SVs, i.e., that occurred in at least two of the three replicates of each compost, were selected.

Compost combinations	number of SVs	Cumulative number of SVs	Percentage of SVs	cumulative percentage of SVs
1	2,447	2,447	16.1	16.1
2	2,635	5,082	17.3	33.4
3	2,279	7,361	15.0	48.3
4	1,807	9,168	11.9	60.2
5	1,366	10,534	9.0	69.1
6	1,041	11,575	6.8	76.0
7	816	12,391	5.4	81.3
8	587	12,978	3.9	85.2
9	478	13,456	3.1	88.3
10	376	13,832	2.5	90.8
11	316	14,148	2.1	92.9
12	243	14,391	1.6	94.5
13	199	14,590	1.3	95.8
14	174	14,764	1.1	96.9
15	148	14,912	1.0	97.9
16	145	15,057	1.0	98.8
17	179	15,236	1.2	100.0

Supplementary Table 8: Effect of disease suppression, growth promotion and compost characteristics on bacterial community structures in different compartements. Bacterial communities were assessed in composts, rhizoplanes grown in different compost substrates and in rhizoplanes including only compost-derived rhizoplane SVs. Bacterial community structures were based on Bray-Curtis dissimilarities using mean relative abundances of robustly detected SVs for each substrate or rhizoplane. For compost H, means across both experiments were used. Dissimilarity based linear models were performed with PERMANOVA including explained variation (expl. var.), pseudo F-statistic (F) and p-value (p).

Factor	Compost communities			Rhizoplane communities derived from compost[a]		
	expl. var.	F	p	expl. var.	F	p
Disease suppression	14.7	2.6	0.0093	10.8	1.8	0.0206
Growth promotion	11.5	1.9	0.0457	9.1	1.5	0.0711
Nitrate	11.6	2.0	0.0419	NA	NA	NA
Disease suppression with nitrate as covariate	9.5	1.7	0.0765	NA	NA	NA
Growth promotion with nitrate as covariate	6.9	1.8	0.2698	NA	NA	NA
Inorganic nitrogen	10.4	1.7	0.0698	NA	NA	NA
age[b]	17.6	2.1	0.0335	NA	NA	NA

NA not available

a for the correlations of rhizoplanes inoculated with *P. ultimum* with mean percent suppression 11,207 SVs derived from composts were used for the calculation of the Bray-Curtis dissimilarity while for the correlations of rhizoplanes without inoculation with *P. ultimum* with mean growth promotion 11,359 SVs were included

b data on age was available for 12 composts

Supplementary Table 9: Partitioning of SVs among five most and least active composts. Mean and standard deviation (in parentheses) of percentage of number of SVs in each area of the Venn diagram between the five compost with least growth promotion (compost F, H, I, K and L) and the five compost with the largest growth promotion (compost D, E, M, N and Q) as well as between the five composts with least (compost A, B, D, E and N) and the five compost with the largest disease suppression against *P. ultimum* (compost H, I, K, L and Q) were presented. Student t-tests were performed and p-values adjusted using the Benjamini Hochberg correction. Menas are also presented in Supplementary Figure 6.

trait	area in Venn	mean and standard deviation of percentage of SVs in		t-test statistic	BH adjusted p-value
		five compost with strongest activity	five composts with least activity		
growth promotion	a1	12.78 (2.69)	12.24 (2.42)	0.35	0.827
	a2	8.5 (1.68)	8.65 (1.6)	-0.15	0.881
	a3	19.71 (3.45)	18.35 (1.98)	0.78	0.771
	a4	0.12 (0.1)	0.15 (0.17)	-0.30	0.827
	a5	0.03 (0.02)	0.04 (0.02)	-0.34	0.827
	a6	0.23 (0.05)	0.26 (0.14)	-0.49	0.827
	a7	4.78 (1.67)	3.49 (1.32)	1.40	0.755
	a8	0.4 (0.27)	0.23 (0.2)	1.14	0.771
	a9	21.06 (5.5)	22.58 (3.74)	-0.52	0.827
	a10	0.05 (0.02)	0.06 (0.02)	-0.82	0.771
	a11	0.07 (0.02)	0.09 (0.02)	-1.46	0.755
	a12	0.94 (0.69)	1.61 (0.74)	-1.56	0.755
	a13	5.45 (1.86)	3.42 (1.23)	2.08	0.755
	a14	11.4 (1.32)	12.08 (0.97)	-0.95	0.771
	a15	14.47 (4.31)	16.75 (3.02)	-1.00	0.771
disease suppression	a1	10.16 (1.66)	7.58 (0.67)	3.48	0.081
	a2	6.43 (0.79)	6.03 (1.42)	0.56	0.810
	a3	22.17 (1.53)	22.4 (3.75)	-0.13	0.903
	a4	0.17 (0.17)	0.05 (0.02)	1.71	0.244
	a5	0.03 (0.02)	0.03 (0.01)	0.15	0.903
	a6	0.29 (0.14)	0.27 (0.06)	0.32	0.903
	a7	3.33 (0.79)	4.62 (1.04)	-2.28	0.118
	a8	0.35 (0.12)	0.71 (0.15)	-4.43	0.038
	a9	22.92 (4.16)	28.44 (3.21)	-2.48	0.098
	a10	0.08 (0.03)	0.13 (0.04)	-2.07	0.145
	a11	0.13 (0.03)	0.08 (0.03)	2.69	0.098
	a12	1.84 (1.06)	0.51 (0.38)	2.84	0.098
	a13	3.52 (1.12)	5.04 (0.98)	-2.41	0.098
	a14	10.65 (0.85)	10.51 (1.78)	0.16	0.903
	a15	17.94 (3.48)	13.6 (5.85)	1.46	0.290

Supplementary Table 10: Relative abundance and taxonomy of SVs associated with growth promoting composts, i.e., D, E, M, N and Q. Potentially growth promoting SVs were selected by comparing robustly detected SVs of the five most and the five least growth promoting composts using indicator species analysis with point biserial correlation coefficient larger than 0.7 as a selection criterion. A literature research was performed to assess if the genera have been mentioned in relation to compost bacteria or plant growth promotion (end October 2020).

			mean sequence abundance [%] in							comparison to literature related to	
SV	R_{PB}[1]	rho / p / n^2	highly growth promoting composts	weakly growth promoting composts	rhizoplanes grown in substrates with high growth promotion	matrix	rhizoplanes grown in the matrix	phylum	lowest possible taxonomic classification2	compost	plant growth promotion
SV-11330	0.84	NA	1.7E-03	0	0	0	0	Proteobacteria	o_Rhizobiales	NA	NA
SV-21591	0.81	NA	8.8E-04	0	3.9E-04	0	0	Proteobacteria	g_Panacagrimonas	No reference	No reference
SV-2745	0.80	NA	1.3E-02	9.3E-04	1.1E-02	0	0	Actinobacteria	c_Actinobacteria	NA	NA
SV-12568	0.78	NA	2.8E-03	2.1E-04	5.5E-04	0	0	Chloroflexi	c_OLB14	NA	NA
SV-4513	0.78	0.23 / 0.38 / 13	2.3E-03	6.9E-04	4.2E-03	0	0	Proteobacteria	g_Microvirga	[1, 2]	[reviewed in 3, 4]
SV-6616	0.77	NA	1.5E-03	0	7.2E-04	0	0	Gemmatimonadetes	f_Gemmatimonadaceae	NA	NA
SV-11998	0.77	NA	1.5E-03	0	1.5E-03	0	0	Proteobacteria	o_Rhizobiales	NA	NA
SV-12378	0.77	NA	5.1E-03	0	1.6E-03	0	0	unclassified	unclassified	NA	NA
SV-5145	0.76	0.66 / 0.03 / 15	7.5E-03	2.2E-03	3.4E-03	0	0	Chloroflexi	c_JG30-KF-CM66	NA	NA
SV-2659	0.75	0.36 / 0.20 / 14	8.0E-03	1.2E-03	2.0E-02	0	0	Actinobacteria	c_Actinobacteria	NA	NA
SV-7153	0.75	NA	1.3E-03	0	2.3E-04	0	0	Proteobacteria	g_Acinetobacter	[5, 6]	[7, 8]
SV-2059	0.74	0.51 / 0.09 / 13	5.6E-03	6.8E-04	2.8E-02	1.5E-02	2.7E-02	Actinobacteria	g_Streptomyces	[9, 10]	[reviewed in 11, 12]
SV-14393	0.74	NA	1.9E-03	0	0	0	0	unclassified	unclassified	NA	NA
SV-10345	0.74	0.3 / 0.29 / 11	1.9E-03	4.9E-04	1.0E-03	0	0	Chloroflexi	o_SAR202_clade	NA	NA
SV-8795	0.73	NA	4.4E-03	0	2.6E-03	0	0	Chloroflexi	c_OLB14	NA	NA
SV-15304	0.73	NA	2.4E-03	4.2E-04	2.3E-04	0	0	Proteobacteria	c_Gammaproteobacteria	NA	NA
SV-1147	0.73	0.69 / 0.03 / 17	3.6E-02	2.0E-02	3.5E-02	0	1.9E-03	Proteobacteria	g_Hyphomicrobium	[13, 14]	No reference
SV-3208	0.73	0.27 / 0.32 / 12	1.2E-02	7.1E-04	5.9E-03	0	0	Acidobacteria	c_Subgroup_6	NA	NA
SV-6142	0.72	NA	2.4E-03	0	2.5E-03	0	0	Gemmatimonadetes	c_S0134_terrestrial_group	NA	NA
SV-9424	0.72	NA	1.9E-03	2.4E-04	3.2E-03	0	0	Proteobacteria	g_Peredibacter	[15]	No reference
SV-31	0.72	0.6 / 0.06 / 13	1.0E-02	2.1E-03	2.6E-01	3.9E+00	1.4E+00	Proteobacteria	g_Bradyrhizobium	No reference	[reviewed in 16, 17]
SV-61	0.72	0.58 / 0.06 / 17	6.6E-01	1.6E-01	1.6E-01	2.1E-03	0	Gemmatimonadetes	c_S0134_terrestrial_group	NA	NA
SV-5197	0.72	0.49 / 0.1 / 13	9.0E-03	2.6E-03	4.1E-04	0	0	Proteobacteria	g_Brevundimonas	[18, 19]	[20]
SV-2097	0.72	0.77 / 0.01 / 14	7.4E-03	1.7E-03	3.2E-02	0	0	Firmicutes	f_Bacillaceae	NA	NA
SV-7098	0.71	NA	3.5E-03	2.4E-04	3.2E-03	0	0	unclassified	unclassified	NA	NA
SV-16762	0.70	NA	1.5E-03	0	1.6E-03	0	0	Proteobacteria	c_Gammaproteobacteria	NA	NA
SV-13305	0.70	NA	1.5E-03	2.3E-04	1.0E-03	6.1E-03	0	Proteobacteria	o_Rhizobiales	NA	NA
SV-4136	0.70	NA	2.2E-03	0	1.6E-02	0	0	Firmicutes	g_Bacillus	[21, reviewed in 22]	[23, 24]

1 point biserial correlation coefficient
2 Spearman correlation of the relative abundance of an SV with mean growth promotion including all 17 composts; with Benjamini Hochberg adjusted p-value.
3 letter preceding the lowest possible taxonomic classification corresponds to the taxonomic rank (g for genus, f for family, o for order, c for class)
NA not available

Supplementary Table 11: Relative abundance, occurrence and taxonomy of 75 SVs associated to strongly suppressive composts, i.e., H, I, K, L and Q. Potentially disease suppressive SVs were selected by comparing robustly detected SVs of the five most and the five least suppressive composts using indicator species analysis with point biserial correlation coefficient larger than 0.7 as a selection criterion. Mean relative sequence abundance of each SV was correlated with mean disease suppression including all 17 composts. For each SV, which was assigned to a genus, evidence from literature about its occurrence in composts and involvement in suppression against soil borne fungal and oomycete diseases was assessed (end October 2020).

SV	R_{PB}[1]	rho / p / n[2]	mean sequence abundance [%] in					phylum	lowest taxonomic classification[2]	comparison to literature related to	
			highly suppr. composts	Weakly suppr. composts	Rhizoplanes of highly suppr. composts + Pu[3]	substrate	Rhizoplanes of substrate + Pu			compost	suppression
SV-437	0.95	0.57 / 0.031 / 16	6.9E-02	8.9E-03	1.0E-01	0	0	Chloroflexi	c_OLB14	NA	NA
SV-48	0.95	0.65 / 0.017 / 17	2.9E-01	6.8E-02	5.8E-01	0	1.9E-03	Proteobacteria	o_Rhizobiales	NA	NA
SV-1095	0.90	0.78 / 0.007 / 17	3.5E-02	4.0E-03	3.6E-02	0	8.2E-03	Proteobacteria	c_Gammaproteobacteria	NA	NA
SV-1471	0.89	0.65 / 0.017 / 17	6.4E-02	9.0E-03	4.2E-02	0	0	Firmicutes	g_Ureibacillus	[25, 26]	No reference
SV-754	0.88	0.55 / 0.035 / 16	5.6E-02	9.3E-03	2.3E-02	0	5.9E-04	Gemmatimonadetes	f_Gemmatimonadaceae	NA	NA
SV-25590	0.88	NA	1.9E-03	0	9.4E-04	0	0	Proteobacteria	c_Gammaproteobacteria	NA	NA
SV-142	0.88	0.53 / 0.04 / 17	1.6E-01	2.7E-02	2.5E-01	0	5.6E-03	Proteobacteria	c_Gammaproteobacteria	NA	NA
SV-2226	0.87	0.64 / 0.017 / 11	1.2E-02	0	1.1E-02	0	0	Firmicutes	g_Ureibacillus	[25, 26]	No reference
SV-6464	0.87	0.68 / 0.013 / 11	2.6E-03	2.0E-04	1.8E-03	0	0	Firmicutes	f_Limnochordaceae	NA	NA
SV-757	0.83	0.4 / 0.112 / 17	3.8E-02	1.2E-02	5.2E-02	0	6.3E-04	Proteobacteria	c_Alphaproteobacteria	NA	NA
SV-11381	0.83	NA	5.8E-03	2.3E-04	4.2E-03	0	0	Planctomycetes	g_Thermogutta	[27]	No reference
SV-458	0.82	0.64 / 0.017 / 17	8.6E-02	1.1E-02	5.3E-02	0	1.2E-03	Firmicutes	g_Ureibacillus	[25, 26]	No reference
SV-23516	0.82	NA	9.5E-04	0	8.3E-04	0	0	Proteobacteria	o_Rhizobiales	NA	NA
SV-2932	0.81	0.64 / 0.017 / 13	4.8E-02	2.9E-03	5.9E-03	0	0	Proteobacteria	c_Gammaproteobacteria	NA	NA
SV-5576	0.80	0.63 / 0.017 / 14	6.8E-03	1.3E-03	2.1E-03	0	0	Bacteroidetes	g_Natronoflexus	No reference	No reference
SV-6245	0.79	NA	2.6E-03	0	3.1E-03	0	0	Firmicutes	f_Thermoactinomycetaceae	NA	NA
SV-162	0.79	0.57 / 0.033 / 17	1.4E-01	1.8E-02	5.7E-02	0	2.9E-03	Proteobacteria	g_Sphingopyxis	[28, 29]	[30, 31]
SV-2898	0.79	NA	2.2E-02	4.9E-04	2.7E-03	0	0	Dadabacteria	o_Dadabacteriales	NA	NA
SV-3619	0.79	NA	2.1E-03	2.2E-04	1.1E-02	0	0	Chloroflexi	f_JG30-KF-CM45	NA	NA
SV-3061	0.78	0.55 / 0.035 / 12	1.7E-02	1.3E-03	7.8E-03	0	0	Gemmatimonadetes	f_Gemmatimonadaceae	NA	NA
SV-64	0.78	0.68 / 0.013 / 14	9.1E-02	5.1E-03	4.4E-02	1.1E-03	6.4E-03	Bacteroidetes	g_Algoriphagus	[32, 33]	No reference
SV-13552	0.78	NA	1.6E-03	0	1.8E-04	0	0	Actinobacteria	g_CL500-29_marine_group	NA	NA
SV-19698	0.78	NA	1.6E-03	0	1.9E-04	0	0	Hydrogenedentes	f_Hydrogenedensaceae	NA	NA
SV-12	0.77	0.64 / 0.017 / 17	1.7E+00	5.5E-01	1.9E+00	4.2E-03	3.6E-03	Chloroflexi	f_A4b	NA	NA
SV-5594	0.77	NA	3.7E-03	2.2E-04	4.5E-03	0	0	Proteobacteria	c_Alphaproteobacteria	NA	NA
SV-1974	0.77	0.83 / 0.003 / 16	1.1E-02	1.6E-03	2.1E-02	0	0	Firmicutes	g_Symbiobacterium	[34, 35]	No reference
SV-1477	0.77	NA	1.4E-02	2.3E-04	1.5E-02	0	0	Gemmatimonadetes	c_S0134_terrestrial_group	NA	NA
SV-5032	0.76	NA	4.5E-03	4.5E-04	2.5E-03	0	0	Firmicutes	f_Limnochordaceae	NA	NA
SV-630	0.76	NA	4.5E-03	2.1E-04	1.3E-02	0	7.0E-02	Proteobacteria	g_Brucella	[36, 37]	No reference
SV-9181	0.76	NA	2.6E-03	0	1.7E-03	0	0	Firmicutes	g_Caldalkalibacillus	[38, 39]	No reference
SV-19508	0.76	NA	1.1E-03	0	0	0	0	Proteobacteria	g_Bdellovibrio	[40, 41]	No reference
SV-13855	0.76	NA	1.1E-03	0	1.6E-03	0	0	Firmicutes	f_Thermoanaerobacterales_Family_III	NA	NA
SV-3867	0.76	NA	7.9E-03	1.1E-03	1.2E-02	0	0	Proteobacteria	o_Rhizobiales	NA	NA
SV-3338	0.76	NA	1.4E-03	0	2.3E-03	0	0	Proteobacteria	f_Xanthomonadaceae	NA	NA

SV-1081	0.76	0.53 / 0.04 / 17	5.5E-02	8.6E-03	1.6E-02	0	0	Proteobacteria	o_Betaproteobacteriales	NA	NA
SV-6670	0.75	NA	8.7E-03	2.3E-04	4.7E-04	0	0	Proteobacteria	g_Salinispirillum	No reference	No reference
SV-6347	0.75	NA	3.1E-03	0	1.6E-03	0	0	Firmicutes	f_Limnochordaceae	NA	NA
SV-1760	0.75	0.59 / 0.025 / 16	1.5E-02	3.2E-03	2.4E-02	0	6.2E-04	Gemmatimonadetes	c_S0134_terrestrial_group	NA	NA
SV-3512	0.75	0.5 / 0.045 / 11	4.5E-03	1.8E-03	7.1E-03	0	0	Proteobacteria	c_Deltaproteobacteria	NA	NA
SV-10781	0.74	NA	2.6E-03	0	1.2E-03	0	0	Bacteroidetes	g_Flavobacterium	[18, 42]	[43, 44]
SV-478	0.74	0.62 / 0.02 / 17	1.4E-01	1.1E-02	3.7E-02	0	0	Proteobacteria	o_Betaproteobacteriales	NA	NA
SV-658	0.74	NA	2.7E-02	4.4E-04	1.9E-02	0	1.1E-02	Verrucomicrobia	g_Luteolibacter	[45]	No reference
SV-133	0.74	0.71 / 0.013 / 12	3.4E-02	7.1E-04	2.6E-01	0	2.3E-01	Proteobacteria	f_Burkholderiaceae	NA	NA
SV-2107	0.74	0.69 / 0.013 / 13	2.7E-02	1.1E-03	9.8E-03	0	0	Planctomycetes	g_Pirellula	[46, 47]	No reference
SV-998	0.73	0.54 / 0.035 / 15	1.1E-01	3.5E-03	2.5E-02	0	0	Planctomycetes	g_Thermostilla	No reference	No reference
SV-3316	0.73	NA	3.9E-03	2.0E-04	1.9E-04	0	0	Bacteroidetes	c_Bacteroidia	NA	NA
SV-529	0.73	0.28 / 0.28 / 16	5.7E-02	2.2E-02	4.1E-02	0	0	Acidobacteria	c_Subgroup_6	NA	NA
SV-5435	0.73	NA	2.4E-03	0	5.3E-03	0	0	Firmicutes	g_Ruminiclostridium	[48, 49]	No reference
SV-23	0.73	0.66 / 0.017 / 17	7.2E-01	2.4E-01	8.2E-01	0	6.3E-04	Chloroflexi	f_A4b	NA	NA
SV-1545	0.73	0.45 / 0.081 / 17	1.9E-02	7.9E-03	1.6E-02	0	1.2E-03	Verrucomicrobia	g_Luteolibacter	[45]	No reference
SV-3440	0.73	0.69 / 0.013 / 13	5.7E-03	4.5E-04	9.5E-03	0	0	Firmicutes	f_Paenibacillaceae	NA	NA
SV-6276	0.73	NA	3.3E-03	0	3.6E-03	0	0	Firmicutes	f_Limnochordaceae	NA	NA
SV-5891	0.73	NA	2.1E-03	0	1.1E-03	4.3E-03	1.8E-03	Bacteroidetes	f_NS11-12_marine_group	NA	NA
SV-1141	0.72	NA	3.1E-02	7.0E-04	6.6E-03	0	0	Bacteroidetes	g_Pedobacter	[50]	[51, 52]
SV-7931	0.72	0.51 / 0.042 / 14	4.2E-03	9.1E-04	6.7E-04	0	0	Proteobacteria	o_Betaproteobacteriales	NA	NA
SV-3861	0.72	NA	4.4E-03	0	2.0E-03	0	0	Bacteroidetes	g_Leadbetterella	[53, 54]	No reference
SV-2995	0.72	0.69 / 0.013 / 13	5.6E-03	2.4E-04	8.8E-03	0	0	Firmicutes	p_Firmicutes	NA	NA
SV-2082	0.72	0.55 / 0.035 / 14	1.4E-02	1.6E-03	1.5E-02	0	0	Proteobacteria	g_Pseudorhodoplanes	No reference	No reference
SV-1543	0.72	0.67 / 0.017 / 15	5.4E-02	6.5E-03	2.0E-02	0	0	Bacteroidetes	f_Chitinophagaceae	NA	NA
SV-260	0.72	NA	1.4E-02	2.0E-04	1.3E-01	0	4.6E-02	Proteobacteria	f_Rhizobiaceae	NA	NA
SV-395	0.72	0.44 / 0.083 / 17	1.3E-01	5.4E-02	3.3E-02	0	0	Proteobacteria	o_R7C24	NA	NA
SV-6873	0.71	NA	5.1E-03	0	5.2E-03	0	1.8E-03	Proteobacteria	f_Burkholderiaceae	NA	NA
SV-11259	0.71	NA	9.9E-03	2.3E-04	4.0E-04	0	0	Proteobacteria	c_Gammaproteobacteria	NA	NA
SV-166	0.71	NA	1.2E-02	1.6E-03	9.4E-02	0	1.7E-02	Proteobacteria	g_Rheinheimera	[32, 55]	[56]
SV-1210	0.71	NA	5.5E-02	6.1E-03	3.2E-02	0	5.8E-04	Verrucomicrobia	g_Diplosphaera	No reference	No reference
SV-10724	0.71	0.56 / 0.035 / 17	1.6E-02	4.8E-03	1.9E-02	0	0	Chloroflexi	f_A4b	NA	NA
SV-1745	0.71	0.23 / 0.365 / 13	4.3E-02	8.6E-03	4.6E-03	0	0	Bacteroidetes	g_Flaviaesturariibacter	No reference	No reference
SV-809	0.71	0.42 / 0.095 / 16	5.4E-02	1.3E-02	2.3E-02	0	0	Verrucomicrobia	f_Verrucomicrobiaceae	NA	NA
SV-2869	0.71	0.64 / 0.017 / 15	2.1E-02	1.1E-03	9.1E-03	0	0	Actinobacteria	g_Thermobispora	[49, 57]	No reference
SV-250	0.71	0.51 / 0.044 / 16	6.8E-02	1.6E-02	9.3E-02	0	4.3E-03	Proteobacteria	f_Devosiaceae	NA	NA
SV-1266	0.70	0.48 / 0.06 / 17	3.7E-02	1.1E-02	1.9E-02	0	0	Proteobacteria	g_Steroidobacter	[58, 59]	[60, 61]
SV-7326	0.70	NA	1.8E-03	0	5.9E-03	0	5.8E-04	Verrucomicrobia	f_Pedosphaeraceae	NA	NA
SV-693	0.70	0.59 / 0.025 / 17	3.4E-02	6.7E-03	5.9E-02	0	0	Actinobacteria	g_Thermopolyspora	[49, 62]	No reference
SV-1220	0.70	NA	3.6E-02	6.1E-04	1.4E-02	0	0	Proteobacteria	o_Rhizobiales	NA	NA
SV-5459	0.70	NA	3.2E-02	9.5E-04	0	0	0	Proteobacteria	f_Solimonadaceae	NA	NA

1 point biserial correlation coefficient
2 Spearman correlation of the relative abundance of an SV with mean growth promotion including all 17 composts; with Benjamini Hochberg adjusted p-value.
3 letter preceding the lowest possible taxonomic classification corresponds to the taxonomic rank (g for genus, f for family, o for order, c for class)
NA not available

Supplementary References

1. Vajna B, Szili D, Nagy A, Márialigeti K: **An improved sequence-aided T-RFLP analysis of bacterial succession during oyster mushroom substrate preparation.** *Microbial Ecology* 2012, **64:**702-713.
2. Chen R, Wang Y, Wei S, Wang W, Lin X: **Windrow composting mitigated CH_4 emissions: Characterization of methanogenic and methanotrophic communities in manure management.** *FEMS Microbiology Ecology* 2014, **90:**575-586.
3. Martínez-Hidalgo P, Hirsch A: **The nodule microbiome: N_2-fixing rhizobia do not live alone.** *Phytobiomes* 2017, **1:**13.
4. Ardley JK, Parker MA, De Meyer SE, Trengove RD, O'Hara GW, Reeve WG, Yates RJ, Dilworth MJ, Willems A, Howieson JG: ***Microvirga lupini* sp. nov., *Microvirga lotononidis* sp. nov. and *Microvirga zambiensis* sp. nov. are alphaproteobacterial root-nodule bacteria that specifically nodulate and fix nitrogen with geographically and taxonomically separate legume hosts.** *International Journal of Systematic and Evolutionary Microbiology* 2012, **62:**2579-2588.
5. Zhu L, Zhao Y, Zhang W, Zhou H, Chen X, Li Y, Wei D, Wei Z: **Roles of bacterial community in the transformation of organic nitrogen toward enhanced bioavailability during composting with different wastes.** *Bioresource Technology* 2019, **285:**121326.
6. Cayuela ML, Mondini C, Insam H, Sinicco T, Franke-Whittle I: **Plant and animal wastes composting: Effects of the N source on process performance.** *Bioresource Technology* 2009, **100:**3097-3106.
7. Rokhbakhsh-Zamin F, Sachdev D, Kazemi-Pour N, Engineer A, Pardesi KR, Zinjarde S, Dhakephalkar PK, Chopade BA: **Characterization of plant-growth-promoting traits of *Acinetobacter* species isolated from rhizosphere of *Pennisetum glaucum*.** *J Microbiol Biotechnol* 2011, **21:**556-566.
8. Kotoky R, Das S, Singha LP, Pandey P, Singha KM: **Biodegradation of Benzo(a)pyrene by biofilm forming and plant growth promoting *Acinetobacter* sp. strain PDB4.** *Environmental Technology & Innovation* 2017, **8:**256-268.
9. Salamoni SP, Mann MB, Campos FS, Franco AC, Germani JC, Van Der Sand ST: **Preliminary characterization of some Streptomyces species isolated from a composting process and their antimicrobial potential.** *World Journal of Microbiology and Biotechnology* 2010, **26:**1847-1856.
10. Kandasamy S, P D, Chendrayan, Uthandi S: **Laccase producing streptomyces bikiniensis CSC12 isolated from compost.** *Journal of microbiology, biotechnology and food sciences* 2016, **6:**794-798.
11. Olanrewaju OS, Babalola OO: **Streptomyces: implications and interactions in plant growth promotion.** *Applied Microbiology and Biotechnology* 2019, **103:**1179-1188.
12. Kamal R, Sharma A: **Control of Fusarium wilt using biological agent Streptomyces sp.CPP-53 isolated from compost with plant growth promoting effect on tomato under greenhouse condition.** *Journal of Microbiology and Antimicrobials* 2014, **6:**97-103.
13. Carrasco J, Tello ML, de Toro M, Tkacz A, Poole P, Pérez-Clavijo M, Preston G: **Casing microbiome dynamics during button mushroom cultivation: implications for dry and wet bubble diseases.** *Microbiology* 2019, **165:**611-624.
14. Tumuhairwe JB, Tenywa J: **Bacterial community changes during composting of municipal crop waste using low technology methods as revealed by 16S rRNA.** *African Journal of Environmental Science and Technology* 2018, **12:**209-221.
15. Munoz-Ucros J, Panke-Buisse K, Robe J: **Bacterial community composition of vermicompost-treated tomato rhizospheres.** *PLOS ONE* 2020, **15:**e0230577.
16. Mehboob I, Naveed M, Zahir ZA: **Rhizobial association with non-Legumes: Mechanisms and applications.** *Critical Reviews in Plant Sciences* 2009, **28:**432-456.
17. Antoun H, Beauchamp CJ, Goussard N, Chabot R, Lalande R: **Potential of *Rhizobium* and *Bradyrhizobium* species as plant growth promoting rhizobacteria on non-legumes: Effect on radishes (*Raphanus sativus* L.).** *Plant and Soil* 1998, **204:**57-67.
18. Sun Y, Men M, Xu B, Meng Q, Bello A, Xu X, Huang X: **Assessing key microbial communities determining nitrogen transformation in composting of cow manure using illumina high-throughput sequencing.** *Waste Management* 2019, **92:**59-67.

19. Vivas A, Moreno B, Garcia-Rodriguez S, Benitez E: **Assessing the impact of composting and vermicomposting on bacterial community size and structure, and microbial functional diversity of an olive-mill waste.** *Bioresource Technology* 2009, **100:**1319-1326.
20. Singh N, Marwa N, Mishra Sk, Mishra J, Verma PC, Rathaur S, Singh N: ***Brevundimonas diminuta* mediated alleviation of arsenic toxicity and plant growth promotion in *Oryza sativa* L.** *Ecotoxicology and Environmental Safety* 2016, **125:**25-34.
21. Lin Y, Du D, Si C, Zhao Q, Li Z, Li P: **Potential biocontrol *Bacillus* sp. strains isolated by an improved method from vinegar waste compost exhibit antibiosis against fungal pathogens and promote growth of cucumbers.** *Biological Control* 2014, **71:**7-15.
22. Lutz S, Thuerig B, Oberhaensli T, Mayerhofer J, Fuchs JG, Widmer F, Freimoser FM, Ahrens CH: **Harnessing the microbiomes of suppressive composts for plant protection: From metagenomes to beneficial microorganisms and reliable diagnostics.** *Frontiers in Microbiology* 2020, **11**.
23. Ghosh S, Penterman JN, Little RD, Chavez R, Glick BR: **Three newly isolated plant growth-promoting bacilli facilitate the seedling growth of canola, *Brassica campestris*.** *Plant Physiology and Biochemistry* 2003, **41:**277-281.
24. Santoyo G, Orozco-Mosqueda MdC, Govindappa M: **Mechanisms of biocontrol and plant growth-promoting activity in soil bacterial species of Bacillus and Pseudomonas: a review.** *Biocontrol Science and Technology* 2012, **22:**855-872.
25. Wang Y, Gong J, Li J, Xin Y, Hao Z, Chen C, Li H, Wang B, Ding M, Li W, et al: **Insights into bacterial diversity in compost: Core microbiome and prevalence of potential pathogenic bacteria.** *Science of The Total Environment* 2020, **718:**137304.
26. Weon HY, Lee SY, Kim BY, Noh HJ, Schumann P, Kim JS, Kwon SW: ***Ureibacillus composti* sp. nov. and *Ureibacillus thermophilus* sp. nov., isolated from livestock-manure composts.** *International Journal of Systematic and Evolutionary Microbiology* 2007, **57:**2908-2911.
27. Zhong XZ, Li XX, Zeng Y, Wang SP, Sun ZY, Tang YQ: **Dynamic change of bacterial community during dairy manure composting process revealed by high-throughput sequencing and advanced bioinformatics tools.** *Bioresource Technology* 2020, **306:**123091.
28. Li M, Xu J, Jiang Z, Li Q: **Molecular understanding of autotrophic CO2-fixing bacterial communities in composting based on RuBisCO genes analysis.** *Journal of Biotechnology* 2020, **320:**36-43.
29. Tian W, Sun Q, Xu D, Zhang Z, Chen D, Li C, Shen Q, Shen B: **Succession of bacterial communities during composting process as detected by 16S rRNA clone libraries analysis.** *International Biodeterioration & Biodegradation* 2013, **78:**58-66.
30. Fujiwara K, Iida Y, Someya N, Takano M, Ohnishi J, Terami F, Shinohara M: **Emergence of antagonism against the pathogenic fungus *Fusarium oxysporum* by interplay among non-antagonistic bacteria in a hydroponics using multiple parallel mineralization.** *Journal of Phytopathology* 2016, **164:**853-862.
31. Klein E, Ofek M, Katan J, Minz D, Gamliel A: **Soil suppressiveness to *Fusarium* disease: Shifts in root microbiome associated with reduction of pathogen root colonization.** *Phytopathology* 2013, **103:**23-33.
32. Cai L, Gong X, Sun X, Li S, Yu X: **Comparison of chemical and microbiological changes during the aerobic composting and vermicomposting of green waste.** *PloS one* 2018, **13:**e0207494-e0207494.
33. Williams LE, Kleinschmidt CE, Mecca S: **Bacterial communities in the digester bed and liquid effluent of a microflush composting toilet system.** *PeerJ* 2018, **6:**e6077-e6077.
34. Rhee SK, Jeon C, Bae JW, Kim K, Song J, Kim JJ, Lee SG, Kim HI, Hong SP, Choi YH, et al: **Characterization of *Symbiobacterium toebii*, an obligate commensal thermophile isolated from compost.** *Extremophiles* 2002, **6:**57-64.
35. Ohno M, Shiratori H, Park MJ, Saitoh Y, Kumon Y, Yamashita N, Hirata A, Nishida H, Ueda K, Beppu T: ***Symbiobacterium thermophilum* gen. nov., sp. nov., a symbiotic thermophile that depends on co-culture with a Bacillus strain for growth.** *International Journal of Systematic and Evolutionary Microbiology* 2000, **50:**1829-1832.
36. Qian X, Sun W, Gu J, Wang XJ, Zhang YJ, Duan ML, Li HC, Zhang RR: **Reducing antibiotic resistance genes, integrons, and pathogens in dairy manure by continuous thermophilic composting.** *Bioresource Technology* 2016, **220:**8.

37. Obi L, Atagana H, Adeleke R, Maila M, Bamuza-Pemu E: **Potential microbial drivers of biodegradation of polycyclic aromatic hydrocarbons in crude oil sludge using a composting technique.** *Journal of Chemical Technology & Biotechnology* 2020, **95:**1569-1579.
38. Chi CP, Chu S, Wang B, Zhang D, Zhi Y, Yang X, Zhou P: **Dynamic bacterial assembly driven by *Streptomyces griseorubens* JSD-1 inoculants correspond to composting performance in swine manure and rice straw co-composting.** *Bioresource Technology* 2020, **313:**123692.
39. Kong W, Sun B, Zhang J, Zhang Y, Gu L, Bao L, Liu S: **Metagenomic analysis revealed the succession of microbiota and metabolic function in corncob composting for preparation of cultivation medium for Pleurotus ostreatus.** *Bioresource Technology* 2020, **306:**123156.
40. Koval SF, Hynes SH, Flannagan RS, Pasternak Z, Davidov Y, Jurkevitch E: ***Bdellovibrio exovorus* sp. nov., a novel predator of *Caulobacter crescentus*.** *International Journal of Systematic and Evolutionary Microbiology* 2013, **63:**146-151.
41. Lei L, Gu J, Wang X, Song Z, Yu J, Wang J, Dai X, Zhao W: **Effects of phosphogypsum and medical stone on nitrogen transformation, nitrogen functional genes, and bacterial community during aerobic composting.** *Science of The Total Environment* 2021, **753:**141746.
42. Kim J-J, Kanaya E, Weon H-Y, Koga Y, Takano K, Dunfield PF, Kwon S-W, Kanaya S: ***Flavobacterium compostarboris* sp. nov., isolated from leaf-and-branch compost, and emended descriptions of *Flavobacterium hercynium*, *Flavobacterium resistens* and *Flavobacterium johnsoniae*.** *International Journal of Systematic and Evolutionary Microbiology* 2012, **62:**2018-2024.
43. Kwok OCH, Fahy PC, Hoitink HJ, Kuter GA: **Interactions between bacteria and *Trichoderma hamatum* in suppression of *Rhizoctonia* damping-off in bark compost media.** *Phytopathology* 1987, **77:**1206-1212.
44. Sang MK, Kim KD: **The volatile-producing *Flavobacterium johnsoniae* strain GSE09 shows biocontrol activity against *Phytophthora capsici* in pepper.** *Journal of Applied Microbiology* 2012, **113:**383-398.
45. Akyol Ç, Ince O, Ince B: **Crop-based composting of lignocellulosic digestates: Focus on bacterial and fungal diversity.** *Bioresource Technology* 2019, **288:**121549.
46. McGee CF, Byrne H, Irvine A, Wilson J: **Diversity and dynamics of the DNA and cDNA-derived bacterial compost communities throughout the Agaricus bisporus mushroom cropping process.** *Annals of Microbiology* 2017, **67:**751-761.
47. De Gannes V, Eudoxie G, Hickey WJ: **Prokaryotic successions and diversity in composts as revealed by 454-pyrosequencing.** *Bioresource Technology* 2013, **133:**573-580.
48. Sukhumavasi J, Ohmiya K, Shimizu S, Ueno K: ***Clostridium josui* sp. nov., a cellulolytic, moderate thermophilic species from Thai compost.** *International Journal of Systematic and Evolutionary Microbiology* 1988, **38:**179-182.
49. Cao G, Song T, Shen Y, Jin Q, Feng W, Fan L, Cai W: **Diversity of bacterial and fungal communities in wheat straw compost for *Agaricus bisporus* cultivation.** *HortScience* 2019, **54:**100-109.
50. Lee H-G, Kim S-G, Im W-T, Oh H-M, Lee S-T: ***Pedobacter composti* sp. nov., isolated from compost.** *International Journal of Systematic and Evolutionary Microbiology* 2009, **59:**345-349.
51. Yin C, Hulbert SH, Schroeder KL, Mavrodi O, Mavrodi D, Dhingra A, Schillinger WF, Paulitz TC: **Role of bacterial communities in the natural suppression of *Rhizoctonia solani* bare patch disease of wheat (*Triticum aestivum* L.).** *Appl Environ Microbiol* 2013, **79:**7428-7438.
52. Song YS, Seo DJ, Jung WJ: **Identification, purification, and expression patterns of chitinase from psychrotolerant *Pedobacter* sp. PR-M6 and antifungal activity in vitro.** *Microbial Pathogenesis* 2017, **107:**62-68.
53. Weon HY, Kim BY, Kwon SW, Park IC, Cha IB, Tindall BJ, Stackebrandt E, Trüper HG, Go SJ: ***Leadbetterella byssophila* gen. nov., sp. nov., isolated from cotton-waste composts for the cultivation of oyster mushroom.** *Int J Syst Evol Microbiol* 2005, **55:**2297-2302.
54. Alessi AM, Bird SM, Oates NC, Li Y, Dowle AA, Novotny EH, deAzevedo ER, Bennett JP, Polikarpov I, Young JPW, et al: **Defining functional diversity for lignocellulose degradation in a microbial community using multi-omics studies.** *Biotechnology for Biofuels* 2018, **11:**166.
55. Evans R, Alessi AM, Bird S, McQueen-Mason SJ, Bruce NC, Brockhurst MA: **Defining the functional traits that drive bacterial decomposer community productivity.** *The ISME Journal* 2017, **11:**1680-1687.

56. Kalinovskaya NI, Romanenko LA, Kalinovsky AI: **Antibacterial low-molecular-weight compounds produced by the marine bacterium *Rheinheimera japonica* KMM 9513T.** *Antonie van Leeuwenhoek* 2017, **110:**719-726.
57. Wang C, Dong D, Wang H, Müller K, Qin Y, Wang H, Wu W: **Metagenomic analysis of microbial consortia enriched from compost: new insights into the role of Actinobacteria in lignocellulose decomposition.** *Biotechnology for Biofuels* 2016, **9:**22.
58. Krishnan Y, Bong CPC, Azman NF, Zakaria Z, Othman NA, Abdullah N, Ho CS, Lee CT, Hansen SB, Hara H: **Co-composting of palm empty fruit bunch and palm oil mill effluent: Microbial diversity and potential mitigation of greenhouse gas emission.** *Journal of Cleaner Production* 2017, **146:**94-100.
59. Yin Y, Gu J, Wang X, Song W, Zhang K, Sun W, Zhang X, Zhang Y, Li H: **Effects of copper addition on copper resistance, antibiotic resistance genes, and intl1 during swine manure composting.** *Frontiers in Microbiology* 2017, **8**.
60. Vida C, Bonilla N, de Vicente A, Cazorla FM: **Microbial profiling of a suppressiveness-induced agricultural soil amended with composted almond shells.** *Frontiers in Microbiology* 2016, **7**.
61. Wang G, Govinden R, Chenia HY, Ma Y, Guo D, Ren G: **Suppression of *Phytophthora* blight of pepper by biochar amendment is associated with improved soil bacterial properties.** *Biology and Fertility of Soils* 2019, **55:**813-824.
62. Zhang X, Zhong Y, Yang S, Zhang W, Xu M, Ma A, Zhuang G, Chen G, Liu W: **Diversity and dynamics of the microbial community on decomposing wheat straw during mushroom compost production.** *Bioresource Technology* 2014, **170:**183-195.
